# Influence of Season, Hive Position, Extraction Method and Storage Temperature on Polyphenols and Antioxidant Activity of Croatian Honey

**DOI:** 10.3390/molecules30040919

**Published:** 2025-02-17

**Authors:** Ivana Šola, Valerija Vujčić Bok, Ivana Fabijanić, Jasna Jablan, Laura Borgese, Andrea Humski, Marina Mikulić, Krešimir Sanković, Zdenko Franić, Gordana Rusak

**Affiliations:** 1 Faculty of Science, Department of Biology, University of Zagreb, Horvatovac 102a, 10000 Zagreb, Croatia; ivana.sola@biol.pmf.unizg.hr; 2 Faculty of Pharmacy and Biochemistry, Department of Pharmaceutical Botany, University of Zagreb, Kovačića 1, 10000 Zagreb, Croatia; 3 Laboratory for Biomolecular Interactions and Spectroscopy, Ruđer Bošković Institute, Bijenička 54, 10000 Zagreb, Croatia; i.s.fabijanic@gmail.com; 4 Faculty of Pharmacy and Biochemistry, Department of Analytical Chemistry, University of Zagreb, Kovačića 1, 10000 Zagreb, Croatia; jasna.jablan@pharma.unizg.hr; 5 INSTM & Chemistry for Technologies Laboratory, Department of Mechanical and Industrial Engineering, University of Brescia, Via Branze 38, 25123 Brescia, Italy; laura.borgese@unibs.it; 6 Laboratory for Food Microbiology, Croatian Veterinary Institute, Savska cesta 143, 10000 Zagreb, Croatia; humski@veinst.hr; 7 Independent Researcher, 10000 Zagreb, Croatia; 8 Faculty of Pharmacy and Biochemistry, Department of Biophysics, University of Zagreb, Kovačića 1, 10000 Zagreb, Croatia; 9 Institute for Medical Research and Occupational Health (IMI), Ksaverska cesta 2, 10000 Zagreb, Croatia; franic@imi.hr

**Keywords:** antibacterial activity, antioxidant activity, *Castanea sativa* honey, flavonoids, heavy metals, phenolic acids, *Robinia pseudoacacia* honey, RP-HPLC

## Abstract

The aim of our study was to compare the composition of polyphenolic compounds between the Croatian acacia (*Robinia pseudoacacia* L.) and chestnut (*Castanea sativa* Mill.) honey from several aspects: production season, hive position (on the edge and in the middle of a series of hives), part of the hive (small or normal extension), and honey extraction method (centrifuging or draining honey combs). Additionally, in acacia honey, we also monitored the influence of different storage temperatures (room temperature (RT) and 4 °C) on the content of phenolic compounds. To separate, identify and quantify individual flavonoids and phenolic acids from the honey, we used the HPLC method. The total polyphenols and antioxidant activity of the samples, their antimicrobial activity and their elemental content were also measured. The significant influence of the season, hive position, and extraction method on the total identified phenolic compounds, phenolic acids, flavonoids, total phenols and antioxidant activity was detected in almost all the acacia and chestnut honey samples. Chestnut honey from 2013 had more total phenolics (TPs) and antioxidant capacity (FRAP) than chestnut from 2014 and 2015. Honey collected from smaller extensions of hives had significantly higher TPs and FRAP compared to normal hive extensions. Centrifugation reduced the TPs and FRAP in most cases, but not always uniformly. Storage at RT led to the predominance of gallic, *p*-coumaric and benzoic acid in acacia honey, while storage at 4 °C maintained *p*-coumaric acid as the dominant phenolic acid. Flavonoids, particularly pinobanksin in acacia honey and hesperetin/pinobanksin in chestnut honey, were less affected by the storage conditions compared to phenolic acids. The non-centrifuged chestnut sample from 2015 showed the lowest MIC values against the most tested pathogenic bacteria. All the honey samples showed an extremely low concentration of heavy metals and relatively high concentrations of potassium and calcium.

## 1. Introduction

Honey is an aqueous solution of various sugars that contains bioactive substances found in the plants from which it originates, which provide it with a distinct nutritional value (polyphenols, vitamins and minerals). Phenolic compounds, specifically flavonoids and phenolic acids, and vitamin C contribute the most to the bioactive properties of honey [1,2,3]. Their synergistic contribution to the antioxidant capacity of honey has been scientifically proven [4,5]. In addition to being used as markers for determining the botanical origin of honey [6,7], flavonoids and phenolic acids could also be used to assess the nutritional and pharmacological value of honey. Namely, numerous studies indicate the extremely positive biological effects of flavonoids and phenolic acids on human health, acting as powerful antioxidants and showing antiviral, antibacterial and anti-inflammatory effects [3,6,7], and analysis of their content would significantly contribute to the nutritional characterization of different types of honey. It is known that hydroxycinnamic acids (caffeic, *p*-coumaric and ferulic acid) are characteristic of chestnut honey [8], the flavonol kaempferol is characteristic of rosemary honey [9], while acacia honey is characterized by kaempferol glycosides [10], ferulic acid and acacetin [11]. Abscisic acid is characteristic of sage honey and naringenin and luteolin of lavender honey [8,9,12]. The content of these compounds in Croatian honey has been very rarely analyzed so far [13,14,15], so these analyses are absolutely necessary and very important for every producer and consumer. Such analyses would not only provide insight into the quality of honey and provide added value with a declaration related to its polyphenolic composition but also enable precise biochemical monitoring of the variations in the nutritional properties of honey that inevitably occur between different honey extraction procedures [16], between different seasons from which honey originates [4,13,17], due to the different ages of honey and the storage conditions, as well as due to the bee species that produced it [2,18]. In addition, such qualitative and quantitative analyses could very effectively test the nutritional value of any imported honey and offer consumers a direct insight into the quality of honey at the level of these compounds.

In 2014, the City Council of Petrinja City, Sisačko-Moslavačka county, adopted a Declaration proclaiming the ecological zone of several villages, including Klinac. The Declaration commits Petrinja City to the promotion of organic farming and protection of natural resources. Klinac is a village of Banija, under the jurisdiction of Petrinja City, located in Sisačko-Moslavačka county, Croatia, at 45°22′29.6″ N and 16°18′27.5″ E. The nature of Klinac is virtually untouched, surrounded by woods, mostly chestnut, which makes it suitable for the promotion of ecological beekeeping and honey production, and the area is now under the supervision of an accredited control body.

The aim of this study was to investigate the composition of polyphenols and minerals, as well as the antioxidant and antibacterial activity, of acacia (*Robinia pseudoacacia* L.) and chestnut (*Castanea sativa* Mill.) honey from Croatia and to estimate the degree of variability of these properties depending on the season, hive position, storage temperature and extraction method. For that purpose, we determined the total phenols and antioxidant activity spectrophotometrically. For the separation, tentative identification and quantification of individual flavonoids and phenolic acids in honey, we used the HPLC method. We analyzed the influence of the hive’s position (on the edge and in the middle of a series of hives) and the part of the hive (small (half-super) or normal extension) on the polyphenol and mineral composition and antioxidant activity of honey. The influence of different storage temperatures (room temperature and 4 °C) on the phenolic composition and antioxidant activity was tested in samples of acacia honey. The influence of normal extraction and centrifuging through nylon (draining of honey combs) on the polyphenol and mineral content and antioxidant activity was monitored in chestnut honey samples. The antibacterial activity against pathogenic Gram-positive and Gram-negative bacterial strains (ATCC and wild type) was tested in selected acacia and chestnut honey samples. The results of this study contribute to the drafting of a declaration on the composition of polyphenols in honey and consumer protection in the Croatian honey market. Such analyses enable producers to single out honey samples of a higher standard, while obtaining a declaration gives the product added value, and consumers are able to choose a Croatian product of known quality.

## 2. Results

### 2.1. RP-HPLC Analysis of Phenolic Acids and Flavonoids

**Figure 1:**
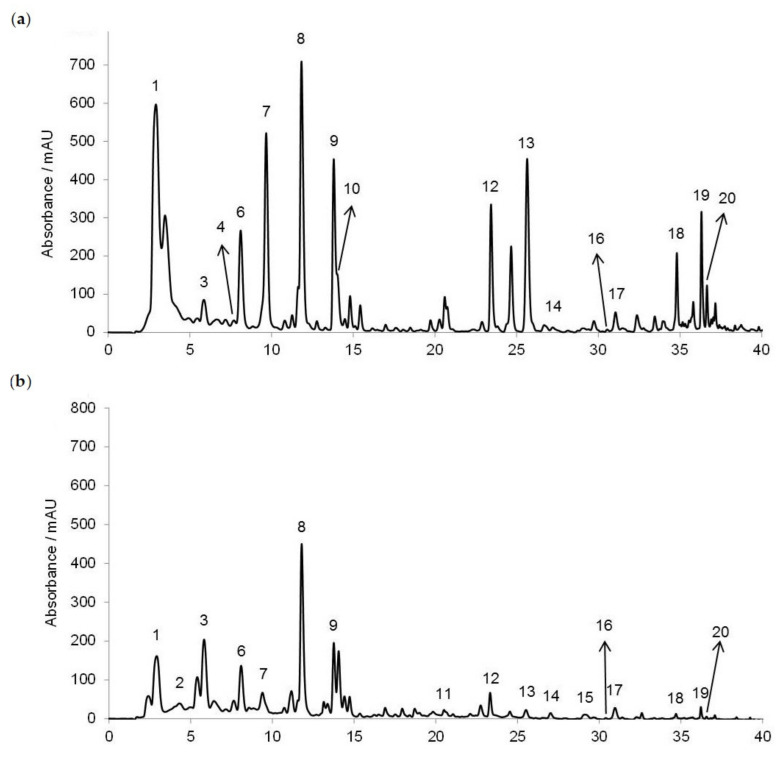
HPLC profiles of the phenolic acids and flavonoids recorded at 280 nm in (**a**) acacia and (**b**) chestnut honey. 1 = gallic acid, 2 = protocatechuic acid, 3 = benzoic acid, 4 = vanillic acid, 5 = chlorogenic acid, 6 = caffeic acid, 7 = syringic acid, caffeine not detected, 8 = *p*-coumaric acid, 9 = ferulic acid, 10 = salicylic acid, 11 = 3′,4′,7-trihydroxyisoflavone (3′-hydroxydaidzein), 12 = cinnamic acid, 13 = pinobanksin, 14 = quercetin, genistein not detected, 15 = hesperetin, 16 = kaempferol, 17 = apigenin, 18 = pinocembrin, 19 = chrysin and 20 = galangin.

The RP-HPLC method was used for the honey polyphenols’ (phenolic acids and flavonoids) separation, tentative identification and quantification (Figure 1). In Croatian acacia honey (Figure 1a), we identified nine phenolic acids (gallic acid, benzoic acid, vanillic acid, caffeic acid, syringic acid, *p*-coumaric acid, ferulic acid, salicylic acid and cinnamic acid) and eight flavonoids (3,4,7-trihydroxyisoflavon, pinobanksin, quercetin, kaempferol, apigenin, pinocembrin, chrysin and galangin), while in chestnut honey (Figure 1b) we identified nine phenolic acids (gallic acid, protocatechuic acid, benzoic acid, vanillic acid, caffeic acid, syringic acid, ferulic acid, salicylic acid and cinnamic acid) and nine flavonoids (3′,4′,7-trihydroxyisoflavone (3′-hydroxydaidzein), pinobanksin, quercetin, hesperetin, kaempferol, apigenin, pinocembrin, chrysin and galangin). Considering the available standards, only genistein and caffeine were not detected in both acacia and chestnut honey.

**Figure 2:**
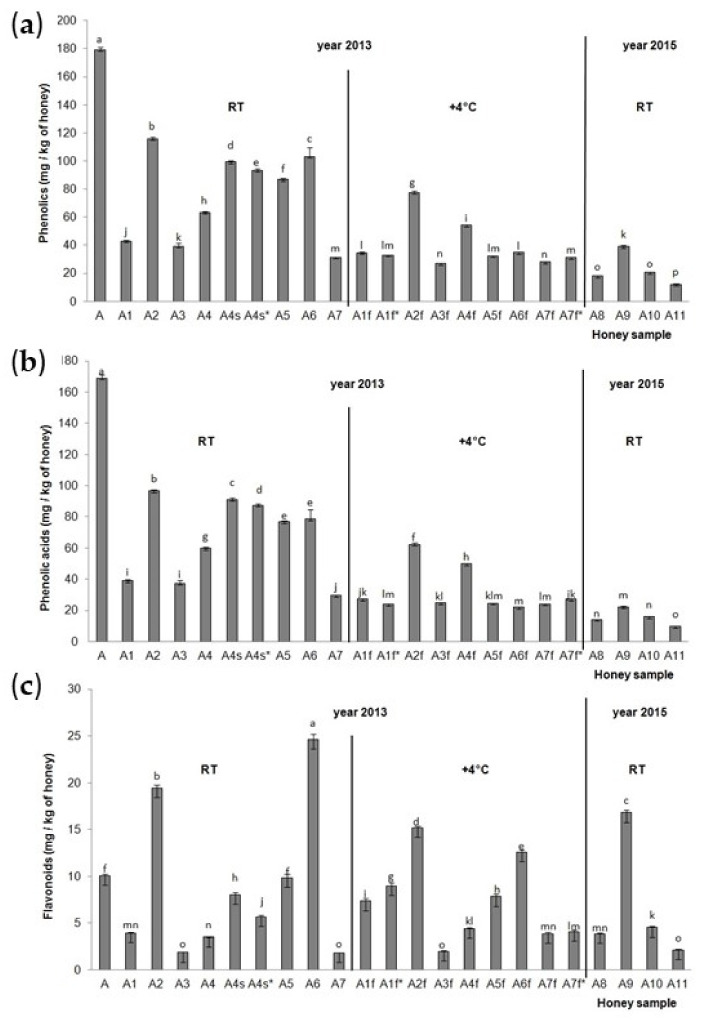
Total identified (**a**) phenolic compounds, (**b**) phenolic acids and (**c**) flavonoids in the Croatian acacia honey stored at room temperature (A1–A7 and A8–A11) after collection in 2013 and 2015 and stored at the low temperature of 4° C (A1f–A7f) after collection in 2013. Values are the mean ± SD based on three replicates. Bars with different letters are significantly different at *p* < 0.05. f = fridge, RT = room temperature, s = small extension = half-super (part of the hive), * = repeated sample triplicate.

The total identified phenolic compounds (TiPs), total identified phenolic acids (TiPAs) and total identified flavonoids (TiFs) for the acacia honey samples were calculated and are presented in Figure 2. The highest TiPs and TiPAs (Figure 2a,b) were detected in acacia aggregation sample A from 2013 stored at RT, and then in acacia sample A2 collected from hive No. 14 from a normal extension (part of the hive) in 2013 and stored at room temperature. The lowest TiPs and TiPAs were in the acacia sample collected from field 5, hive No. 5 in 2015 (A11) and stored at room temperature. All the acacia honey samples stored at room temperature (A1–A7) had higher TiP and TiPA values than the acacia honey samples stored at the low temperature of 4 °C (A1f–A7f), with the exception of the A7f* sample. For TiFs (Figure 2c), significantly lower values were observed in A2f, A5f, A6f compared to A2, A5, A6, and significantly higher values were observed in A1f, A1f*, A7f and A7f* compared to A1 and A7. Significant decreases in TiPs, TiPAs and TiFs were observed in A4 (acacia sample collected from hive No. 32, and from a normal extension of the hive) than A4s and A4s* (acacia sample collected from hive No. 32 and from a small extension of the hive). The position of the hive (on the edge and in the middle of a series of hives) also had a significant influence on the TiP, TiPA and TiF content in the acacia honey samples. Acacia honey samples A2 and A5 (in the middle of a series of hives) had higher TiP, TiPA and TiP values than acacia honey sample A3 collected at the end of a series of hives. The significant influence of the honey extraction method was observed; namely, the centrifuged acacia honey sample A7 had lower concentrations of TiPs, TiPAs and TiFs than the non-centrifuged, i.e., pressed, acacia honey samples (A2–A6) with the exception of the A3 sample for the TiFs (it had a similar concentration to the sample A7). The highest accumulation of TiFs was detected in acacia honey samples A6 and A2 from 2013 and stored at RT, and in acacia honey sample A9 from 2015 stored at RT, while the lowest was in the samples A3 and A7 from 2013 stored at RT, the A3f sample from 2013 stored at a low temperature, and the A11 sample from 2015 stored at RT. The acacia aggregation honey sample from 2013 stored at RT (A1) had significantly higher values of TiPs and TiPAs than the acacia aggregation honey sample from 2015 stored at RT (A8). There was no significant difference between these two samples in terms of the concentration of TiFs. Aggregation sample A from 2013 had significantly higher values of TiPs, TiPAs and TiFs than aggregation sample A8 from 2015.

The most common phenolic acids and flavonoids in the acacia honey samples are presented in Table 1 and Table 2. In acacia aggregation sample A stored at RT from 2013, the most common phenolic acids were gallic acid, *p*-coumaric acid and ferulic acid, and the most common flavonoids were pinobanksin, pinocembrin, apigenin and chrysin. The acacia sample collected from hive No. 14 from a normal extension (part of the hive) in 2013 and stored at RT (A2) had *p*-coumaric acid, gallic acid and chlorogenic acid as the predominant phenolic acids, and pinobanksin, galangin and chrysin as the predominant flavonoids. Gallic acid, chlorogenic acid and syringic acid were the most common phenolic acids, and pinobanksin, pinocembrin and galangin were the most common flavonoids, in acacia honey sample A6 from 2013 stored at RT. Chlorogenic acid, *p*-coumaric acid, ferulic acid and benzoic acid were the main phenolic acids and pinobanksin, pinocembrin and galangin were the main flavonoids in acacia honey sample A9 from 2015 stored at RT. Acacia aggregation honey sample from 2013 stored at RT (A1) had gallic acid, syringic acid and *p*-coumaric acid as the main phenolic acids, while acacia aggregation sample from 2015 stored at RT (A8) had chlorogenic acid, *p*-coumaric acid and benzoic acid as the predominant ones. Both aggregation samples had the same main flavonoids (pinobanksin, chrysin and pinocembrin).

**Figure 3:**
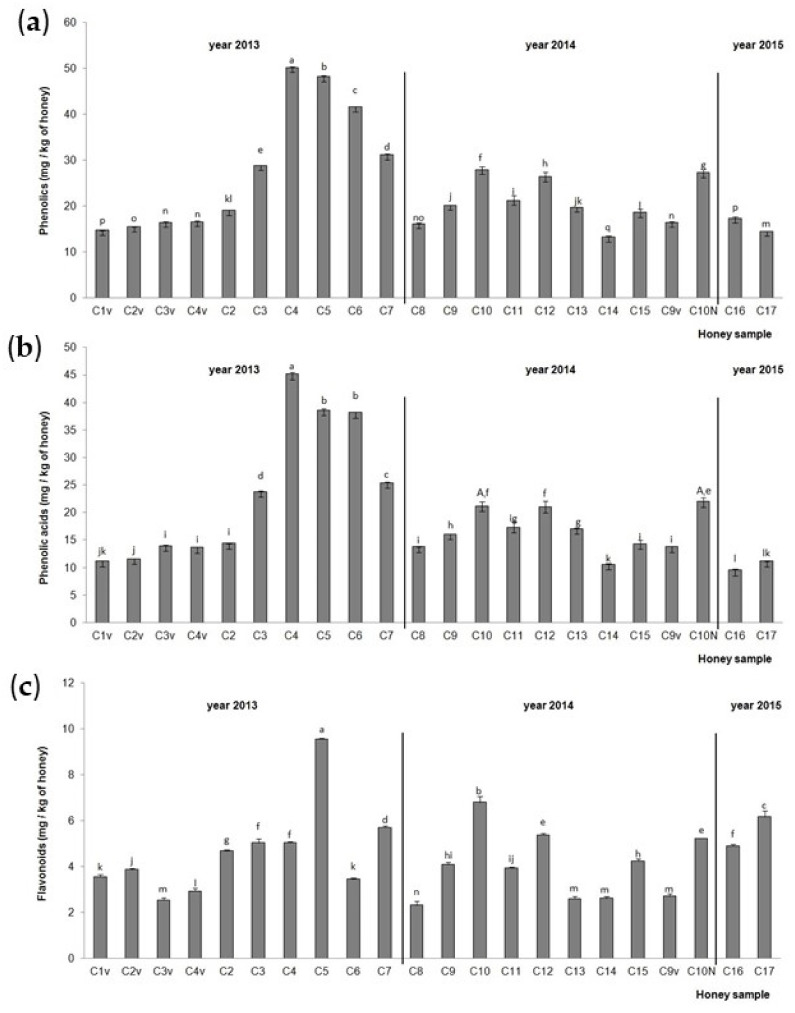
Total identified (**a**) phenolic compounds, (**b**) phenolic acids and (**c**) flavonoids in the Croatian chestnut honey stored at room temperature (C1–C17) after collection in 2013, 2014 and 2015. Values are the mean ± SD based on three replicates. Bars with different letters are significantly different at *p* < 0.05. N = normal extension (part of the hive), v = centrifuged.

The TiPs, TiPAs and TiFs in the chestnut honey samples are presented in Figure 3. The highest TiPs and TiPAs (Figure 3a,b) were detected in chestnut honey samples C4 (hive No. 23, NCF), C5 (hive No. 14, NCF) and C6 (hive No. 36, NCF) from 2013, and the lowest in C14 (hive No. 1, NCF) from 2014 for TiPs and C16 aggregation (RT) sample from 2015 for TiPAs. Samples C5 (hive No. 14, NCF) from 2013, C10 (hive No. 4, NCF) from 2014 and C17 (hive No. 5, CF) from 2015 had the highest TiF (Figure 3c) values. The lowest TiF value was recorded in the aggregation (RT) sample from 2014 (C8). Chestnut honey sample C10 collected from a small extension in 2014, and stored at RT, had higher TiP and TiF values than chestnut honey sample C10N collected from a normal extension in 2014 and stored at RT. Higher TiPA values were noticed in sample C10N from the normal hive extension than in chestnut honey sample C10 from a hive small extension when the statistics were compared for all the chestnut samples together. When the statistics were compared between these two samples only, no significant difference was observed. A significant increase in TiPs, TiPAs and TiFs was observed in the hives in the middle of a series of hives (samples C3, C4, C5, C6) compared to the hive on the edge of a series of hives (samples C2 and C7), with the exception of the C6 and C7 samples for TiFs. The centrifuged chestnut honey samples (C2v, C3v and C4v) from 2013 and C9v from 2014 had lower values of TiPs, TiPAs and TiFs compared to the non-centrifuged chestnut honey sample (C2, C3, C4 and C3) from 2013 and C9v from 2014. The chestnut aggregation honey sample from 2015 (C16) had significantly higher values of TiPs and TiFs compared to the chestnut aggregation honey samples from 2013 (C1v) and 2014 (C8), while the chestnut aggregation honey sample from 2014 (C8) had the highest value of TiPAs.

The most common phenolic acids and flavonoids in the chestnut honey samples are presented in Table 3 and Table 4. The non-centrifuged chestnut honey sample C4 had protocatechuic acid, ferulic acid and benzoic acid as the most common phenolic acids, and hesperetin, pinobanksin and 3′,4′,7-trihydroxyisoflavone (3′-hydroxydaidzein) as the most common flavonoids. In the non-centrifuged chestnut honey sample C5, the main phenolic acids were ferulic, syringic and vanillic acid, and the flavonoids were hesperetin, pinocembrin and 3′-hydroxydaidzein. C4v (nylon extracted) had protocatechuic acid, ferulic acid and vanillic acid as the predominant phenolic acids, and hesperetin, 3′-hydroxydaidzein and Pbs were the predominant flavonoids, while the C3v had ferulic acid, protocatechuic acid and vanillic acid as the main phenolic acids, and hesperetin, 3′-hydroxydaidzein and kaempferol as the main flavonoids. The chestnut aggregation honey samples from 2013 (C1v) and from 2014 (C8) had ferulic acid, protocatechuic acid and vanillic acid as the main phenolic acids, while the aggregation sample from 2015 (C16) had ferulic acid, vanillic acid and benzoic acid as the main phenolic acids. Differences between these three aggregation samples were observed in terms of the main flavonoids (C1v: hesperetin, kaempferol, Pbs, C8: Pbs, hesperetin and kaempferol, C16: hesperetin, chrysin, Pbs).

### 2.2. Spectrophotometric Determination of Total Phenols and Antioxidant Activity

The total phenolic content determined by the Folin–Ciocalteu method and the antioxidant activity determined using the FRAP method of the Croatian acacia and chestnut honey are presented in Table 5.

The highest TP and FRAP values were detected in the non-centrifuged acacia honey sample A5 collected from a normal extension (part of the hive) of hive No. 22 in 2013, while the lowest TP value was in the acacia honey aggregation sample from 2013 (A1), and the lowest FRAP values were in the acacia honey aggregation sample from 2015 (A8) and the non-centrifuged acacia honey sample A9 collected from hive No. 3 in 2015. All the acacia honey samples stored at room temperature (A1–A7) in 2013 had lower TP and FRAP values than the acacia honey samples stored at the low temperature of 4 °C (A1f–A7f) in the same season, with the exception of A5 and A6, which had significantly higher TP and FRAP values than the A5f and A6f samples. A significant decrease in TPs and FRAPs was observed in A4 (acacia sample collected from a normal extension of hive No. 32) compared to A4s (acacia sample collected from a small extension of hive No. 32). Acacia honey samples A2 and A5 (in the middle of a series of hives) had higher TP and FRAP values than acacia honey sample A3 collected at the end of a series of hives. The centrifuged acacia honey sample A7 had lower concentrations of TPs and FRAPs compared to the non-centrifuged acacia honey samples (A2–A6), with the exception of the A4 sample for TPs and A3 and A4 for FRAPs. The acacia aggregation (RT) honey sample from 2013 (A1) had significantly higher values of FRAPs and significantly lower TP values than the acacia aggregation (RT) honey sample from 2015 (A8). In the chestnut honey samples, the highest FRAP values were detected in the C1v aggregation honey sample from 2013 and then in the C10 non-centrifuged sample collected from a small extension (part of the hive) in 2014 and stored at RT. The highest TP values were detected in C10 and then in the C1v sample. The lowest values for both methods were recorded for the centrifuged (nylon) sample C4v from 2013. Sample C10 collected from a small extension had significantly higher TP and FRAP values than C10 N collected from a normal extension. A significant increase (samples C3 and C6) and decrease (C4 and C5) in TPs and FRAPs were observed in the hives in the middle of a series of hives compared to the hives on the edge of a series of hives (samples C2 and C7), with the exception of the C6 and C7 samples for FRAPs. No trend could be observed between the centrifuged and non-centrifuged chestnut honey samples. Sample C4v (centrifuged through nylon) had significantly lower values of TPs and FRAPs than the C3v (normal centrifuging) honey sample. The aggregation honey sample from 2013 (C1v) had significantly higher values of TPs and FRAPs compared to the chestnut aggregation honey samples from 2014 (C8) and 2015 (C16).

### 2.3. Antibacterial Activity

Ten selected acacia and chestnut honey samples were tested for their antimicrobial activity against pathogenic Gram-positive and Gram-negative bacteria (Table 6).

The MIC values expressed as the % (*w*/*v*) of honey varied from 1.56 to 50%. The lowest MIC value (1.56%) was observed in chestnut sample C17 from 2015 against *B. cereus* ATCC 11778. A low MIC value (3.13%) was observed for the samples A9, C9v and C16, and for A8, C4v, C4 and C8 (6.25%) against the same strain. An MIC value of 6.25% against *Y. enterocolitica* ATCC 23715 was observed for the C16 and C17 honey samples, against *Y. enterocolitica*—food isolate and *S. aureus*—food isolate for the C8, C9v, C16 and C17 honey samples and against *S. aureus* ATCC 25923 for the C8, C16 and C17 honey samples. Regarding the antibacterial activity of honey, the chestnut sample C17 (hive No. 5, CF) from 2015 showed the highest overall effectiveness.

### 2.4. Element Content Determined by Total Reflection X-Ray Fluorescence (TXRF)

The Croatian acacia and chestnut honey element content is presented in Table S1. The main elements in the acacia honey samples were K, Ca and P, while the lowest were Ni and Pb. S and Sr were not detected in any of the acacia honey samples. The highest concentrations of K and Ca were detected in the centrifuged acacia honey sample A7 from the year 2013. The non-centrifuged acacia honey sample A11 from 2015 had the lowest values of P, S, Cl, K and Zn. Higher values of K and Ca were measured in the centrifuged (A7) compared to the non-centrifuged (A1–A6) acacia honey samples. In the chestnut honey samples, the main elements were K, Ca and Mn. Ni, Sr, Cr and Pb were the lowest elements detected in the chestnut honey samples. The highest concentration of K was detected in the non-centrifuged chestnut honey sample C2 from 2013, and the highest concentration of Ca was detected in the centrifuged sample C17 from the year 2015. The non-centrifuged chestnut honey sample C6 from 2013 had the lowest values of Cl, Rb, Mn and K. Higher values of K and Ca were measured in the non-centrifuged (C2, C3, C4) compared to the centrifuged (C2v, C3v, C4v) chestnut honey samples. Sample C4v centrifuged through nylon had lower values of K and Ca than the normal centrifuged sample (C3v).

### 2.5. Pearson’s Correlation Coefficients and PCA

The Pearson’s correlation coefficients between the polyphenolic content and the antioxidant activity (FRAP) of Croatian acacia and chestnut honey are presented in Table S2. In the acacia honey samples, the TiPs correlated very strongly (r > 0.80) with the TiPAs (0.99), chlorogenic acid (0.87) and chrysin (0.80), or strongly (r > 0.60 < 0.79) with the TiFs (0.72), gallic acid (0.71) syringic acid (0.64), 4-coumaric acid (0.77), ferulic acid (0.74), Pbs (0.65), kaempferol (0.65), apigenin (0.65) and galangin (0.66). The TiPAs correlated very strongly with chlorogenic acid (0.80) and strongly with gallic acid (0.77), syringic acid (0.60), 4-coumaric acid (0.77), ferulic acid (0.73) and chrysin (0.74). The TiFs correlated very strongly with chlorogenic acid (0.87), Pbs (0.97), kaempferol (0.80), apigenin (0.96), pinocembrin (0.91) and galangin (0.98), and strongly with syringic acid (0.60) and chrysin (0.74). The TPs correlated very strongly with the antioxidant activity (FRAP; 0.99). The majority of individual phenolics in the acacia honey significantly correlated among themselves. Among the individual phenolics, chlorogenic acid correlated very strongly with *p*-coumaric acid (0.80), Pbs (0.85), kaempferol (0.88), apigenin (0.88) and galangin (0.83), and strongly with syringic acid (0.61), ferulic acid (0.78), pinocembrin (0.72) and chrysin (0.65). *p*-coumaric acid correlated very strongly with ferulic acid (0.98) and strongly with kaempferol (0.73). Pbs correlated very strongly with apigenin (0.96), pinocembrin (0.81) and galangin (0.93), and strongly with kaempferol (0.74) and chrysin (0.61). K correlated very strongly with apigenin (0.86), pinocembrin (0.81) and galangin (0.83), and apigenin correlated very strongly with pinocembrin (0.86) and galangin (0.95). Pinocembrin correlated very strongly with galangin (0.95) and strongly with chrysin (0.69). Gallic acid correlated strongly with chrysin (0.61), syringic acid correlated strongly with Pbs (0.69), ferulic acid correlated strongly with kaempferol (0.76), pinocembrin correlated strongly with chrysin (0.69) and chrysin correlated strongly with galangin (0.70). In the chestnut honey samples, the TiPs correlated very strongly (r > 0.80) with the TiPAs (0.99) or strongly (r > 0.60 < 0.79) with the TiFs (0.60), protocatechuic acid (0.71), benzoic acid (0.73), vanillic acid (0.62), syringic acid (0.77) and ferulic acid (0.61). The TiPAs correlated strongly with protocatechuic acid (0.76), benzoic acid (0.70) and syringic acid (0.73). The TiFs correlated very strongly with pinocembrin (0.80) and strongly with vanillic acid (0.60), syringic acid (0.63), apigenin (0.75), chrysin (0.76) and galangin (0.76). The TPs correlated very strongly with the antioxidant activity (FRAP; 0.96). The smaller amount of individual phenolics in the chestnut acacia honey significant correlated among themselves compared to the acacia honey. Among the individual phenolics, vanillic acid correlated very strongly with syringic acid (0.94) and strongly with apigenin (0.62) and pinocembrin (0.66). Pbs correlated very strongly with galangin (0.85) and strongly with chrysin (0.63). Apigenin correlated very strongly with pinocembrin (0.91) and strongly with galangin (0.66). Chrysin correlated very strongly with galangin (0.87). Benzoic acid correlated strongly with vanillic acid (0.71) and syringic acid (0.66), syringic acid correlated strongly with apigenin (0.63) and pinocembrin (0.71), 3′-hydroxydaidzein correlated strongly with cinnamic acid (0.62) and pinocembrin correlated strongly with chrysin (0.72) and galangin (0.79). A strong negative Pearson’s correlation was observed between kaempferol and pinocembrin (−0.64).

The Pearson’s correlation coefficients between the detected elements in Croatian honey are presented in Table S3. A very strong correlation was observed between P and Cr (0.92), between P and Fe (0.86), between P and Cu (0.94), between Cl and K (0.90), between Cl and Ca (0.80), between K and Rb (0.84), between Cr and Fe (0.93), between Cr and Cu (0.94), and between Fe and Cu (0.89). A strong positive correlation was observed between Cl and Rb (0.70), between K and Ca (0.78), and between Ca and Rb (0.69), while a strong negative correlation was observed between Zn and Ga (−0.63) in the acacia honey samples (Table S6A). Between S and Cr (0.84), Cr and Fe (0.91), and Mn and Rb (0.96) was observed a very strong correlation in the chestnut honey samples (Table S6B). In the same samples, strong correlations were observed between S and Fe (0.73), Cl and Ca (0.68), K and Mn (0.76), K and Rb (0.63), Fe and Zn (0.69), and between Rb and Sr (0.77).

**Figure 4:**
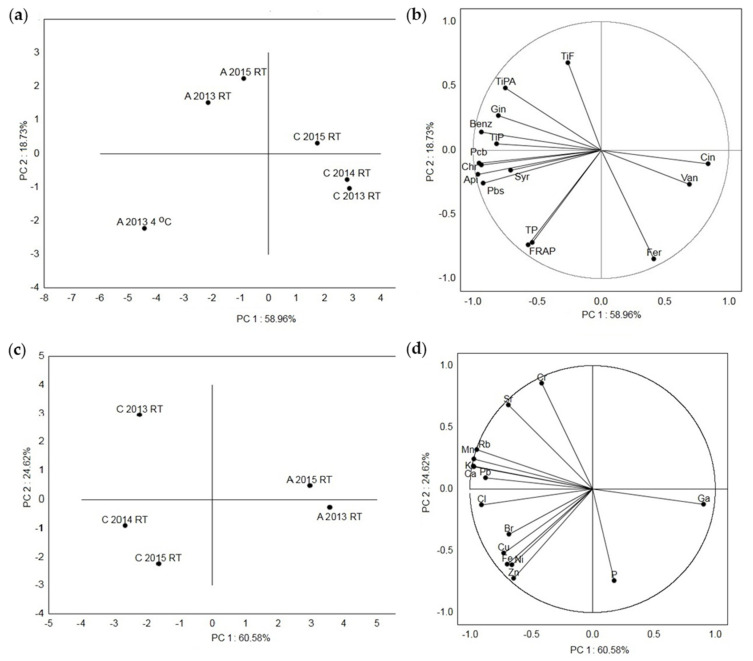
Principal component analysis of the measured metabolites and elements in the Croatian acacia honey stored at room temperature (A 2013 RT and A 2015 RT) after collection in 2013 and 2015 and at the low temperature of 4 °C (A 2013 4 °C), and in the Croatian chestnut honey stored at room temperature after collection in 2013, 2014 and 2015 (C 2013 RT, C 2014 RT, C 2015 RT). (**a**) Score plot separating the acacia and chestnut honey samples at RT and at 4 °C, (**b**) loading plot of the metabolites as variables, (**c**) score plot separating the acacia and chestnut honey samples at RT, and (**d**) loading plot of the elements as variables. Api = apigenin, Benz = benzoic acid, Chr = chrysin, Cin = cinnamic acid, Fer = ferulic acid, FRAP = ferric-reducing/antioxidant power assay, Gin = galangin, Pbs = pinobanksin, Pcm = pinocembrin, Syr = syringic acid, TiFs = total identified flavonoids, TiPs = total identified phenolic compounds, TiPAs = total identified phenolic acids, TPs = total phenols, Van = vanillic acid.

In Figure 4, the principal component analysis (PCA) of the acacia and chestnut honey samples based on their metabolic content and antioxidant capacity (Figure 4a,b), as well as their elemental composition (Figure 4c,d), is shown. The first (PC 1) and the second (PC 2) principal components described 58.96% and 18.73%, respectively, of the variance for the Croatian honey samples based on their polyphenol composition and antioxidant activity (Figure 4a,b). The highest distance was detected between all the acacia and chestnut samples, acacia at a low temperature (A 2013 4 °C) and acacia stored at RT (A 2013 RT and A 2015 RT) and between the chestnut samples C 2015 RT and the samples from 2014 and 2015 (C 2013 RT and C 2014 RT). The smallest distance was detected between the C 2013 RT and C 2014 RT samples and between A 2013 RT and A 2015 RT. Sample A 2013 4 °C had strong loadings with the tested TPs, individual (Pbs, syringic acid, apigenin, chrysin and Pcb) compounds and antioxidant (FRAP) activity, samples A 2013 RT and A 2015 RT had strong loadings with the TiPs, TiPAs, TiFs and individual (benzoic acid and galangin) compounds and samples C 2013 RT and C 2014 RT had strong loadings with ferulic acid, vanillic acid and cinnamic acid. The first (PC 1) and the second (PC 2) principal components described 60.58% and 24.62% of the variance based on the measured elements (Figure 4c,d). Among all the samples, the highest distance was detected between two chestnut samples (C 2014 RT and C2015 RT) and the chestnut sample C 2013 RT. The smallest distance was detected between the C 2015 RT and C 2014 RT samples and between A 2013 RT and A 2015 RT. To the separation of sample C 2013 RT, the elements Cr, Sr, Rb, Mn, K, Ca, and Pb contributed the most. The separation of samples C 2014 RT and C 2015 RT was mostly due to the elements Cl, Br, Cu, Fe, Ni and Zn, and sample A 2013 RT had strong loadings with the elements P and Ga.

## 3. Discussion

### 3.1. Polyphenols and Antioxidant Activity

Using the RP-HPLC method, we identified nine phenolic acids (gallic acid, benzoic acid, vanillic acid, caffeic acid, syringic acid, *p*-coumaric acid, ferulic acid, salicylic acid and cinnamic acid) and eight flavonoids (3′,4′,7-trihydroxyisoflavone, pinobanksin, quercetin, kaempferol, apigenin, pinocembrin, chrysin and galangin) in Croatian acacia honey (Figure 1a), and nine phenolic acids (gallic acid, protocatechuic acid, benzoic acid, vanillic acid, caffeic acid, syringic acid, ferulic acid, salicylic acid and cinnamic acid) and nine flavonoids (3′,4′,7-trihydroxyisoflavone, pinobanksin, quercetin, hesperetin, kaempferol, apigenin, pinocembrin, chrysin and galangin) in Croatian chestnut honey (Figure 1b). Most of the identified phenolic acids and flavonoids for both monofloral Croatian honey types were in accordance with the results of other authors [3,8,10,11,13,19]. The total phenols (TPs), total identified phenolic compounds (TiPs), total identified phenolic acids (TiPAs), total identified flavonoids (TiFs) and antioxidant activity were higher for the acacia than the chestnut honey samples, and they were in the range 129.62–3329.8 mg GAE/g DW, 26.99–115.92 mg/kg, 9.91–169.15 mg/kg, 1.82–24.60 mg/kg, and 1320.73–27642.13 μM Fe(II)/g DW for the acacia honey (Figure 2, Table 1), and 56.77–320.32 mg GAE/g DW, 13.23–50.21 mg/kg, 9.58–45.16 mg/kg, 2.32–9.56 mg/kg and 516.63–2778.16 μM Fe(II)/g DW for the chestnut honey (Figure 4, Table 1), respectively. The amounts quantified in our study were in the same range or higher than those in Romanian acacia honey [19] and Croatian acacia and chestnut honey analyzed in previous studies [13,15,20]. However, Piljac-Žegarac et al. [20] recorded higher TP and FRAP values for chestnut compared to acacia honey from the collection season 2007. This discrepancy might be attributed to the season of the honey collection, hive position (on the edge or in the middle of a series of hives), hive part (small or normal), extraction method (centrifuging or draining honey combs) and storage temperature (room temperature and 4 °C). Since in the work of Piljac-Žegarac et al. [20], besides the collection season, there are no more details on the collection of honey, we cannot draw a parallel with our work. We detected the significant influence of the season on almost all the polyphenol and antioxidant parameters in the acacia and chestnut honey samples, which is in accordance with the results of other authors [4,13,17]. The acacia honey from 2013 was a better source of TiPs, TiPAs and antioxidants, according to the FRAP measurement, compared to 2015. In this honey sample from 2013, the most common phenolic acids were gallic acid, *p*-coumaric acid and ferulic acid, and the most common flavonoids were pinobanksin, pinocembrin, apigenin and chrysin, so we propose that these contributed the most to the antioxidant activity of the sample. The chestnut honey from 2013 was a better source of TPs and antioxidants, according to the FRAP measurement, compared to 2014 and 2015. In the chestnut aggregation sample from 2013, the main phenolic acids were ferulic acid, protocatechuic acid and vanillic acid, and the main flavonoids were hesperetin, kaempferol and pinobanksin.

The significant influence of the hive position (on the edge or in the middle of a series of hives) and hive part (small or normal) on the polyphenols and FRAPs were also observed in both the acacia and chestnut honey samples. The acacia honey samples from in the middle of a series of hives had higher TiP, TiPA, TiF, TP and FRAP values than the acacia honey sample collected from the end of a series of hives. The chestnut honey samples showed the same trend with the most polyphenol parameters. In both honey types, significantly higher values of TiPs, TiPAs, TiFs, TPs and FRAPs were observed in the samples collected from the half-super (small part of hive) compared to the normal part of the hive, with the exception of the chestnut honey samples, where no significant difference was observed for the TiPAs. The small extension of the hive is used to collect honey rather than the large extension of the hive in which the bees are retained. These two extensions differ in their dimensions, bee retention, wax age, and temperature. According to the available literature, these are the first results on the impact of the hive position on the polyphenol content and antioxidant activity. The variation in honeybee foraging behavior could be a potential factor in these differences. The bees may have brought more nectar and pollen to the hives located in the middle. The drifting behavior of bees may be a contributing factor, but this calls for further research.

Ethe extraction method (centrifuging or draining honey combs) used for acacia and chestnut honey also had a significant influence on the polyphenol content and antioxidant activity. The non-centrifuged acacia honey samples had higher values of TiPs, TiPAs, TiFs, TPs and FRAPs than the centrifuged samples. This trend was also observed for the most polyphenol parameters in the chestnut honey samples. Kadri et al. [16] recorded higher values of total carbohydrates, lipids, proteins, flavonoids, ascorbic acid and most minerals in pressed honey due to the greater quantity of pollen. This is in correspondence with our results. Centrifuging through nylon resulted in significantly lower TP and FRAP values compared to normal centrifuging, which means that nylon presented a barrier to the passage of pollen that could affect the higher TiP values and antioxidant activity. The opposite trend was observed for TiPs.

The influence of the storage temperature (RT and 4 °C) on the phenolic composition and antioxidant activity was monitored in the acacia honey samples. Almost all the samples stored at room temperature had higher TiP and TiPA values than the samples stored at 4 °C. The opposite trend was observed for the TPs and FRAPs, and for the TiFs, no trend could be observed. According to Wang et al. [21], low temperature (4 °C) did not significantly affect the antioxidant activity (ORAC) of clover and buckwheat honey; however a significant decrease in TPs was observed. Higher temperatures may influence antioxidants’ stability due to the higher hydroperoxide decomposition, higher reactivity of transition metal ions, and greater rates of general redox reactions [21,22,23]. The differences in the phenolic composition and antioxidant activity among our samples may be due to the different representation of the thermo-stable (polyphenols) and thermo-sensitive (β-carotene, tocopherol, vitamin C) antioxidants in honey. Namely, the TiP, TiPA and TiF values in acacia honey were calculated based on nine identified phenolic acids (gallic acid, benzoic acid, vanillic acid, caffeic acid, syringic acid, *p*-coumaric acid, ferulic acid, salicylic acid and cinnamic acid) and eight identified flavonoids (3′,4′,7-trihydroxyisoflavone, pinobanksin, quercetin, kaempferol, apigenin, pinocembrin, chrysin and galangin) that show different thermostability levels.

### 3.2. Antibacterial Activity

The activity of selected acacia and chestnut honey samples against the pathogenic Gram-positive reference strains *B. cereus* ATCC 11778, *S. aureus* ATCC 25923, and *L. monocytogenes* ATCC 13932 and the Gram-negative reference strains *E. coli* ATCC 25922, *S. enteritidis* ATCC 13076, *P. aeruginosa* ATCC 27583, *Y. enterocolitica* ATCC 23715 and their food isolates was tested. The range of MIC values (1.56–50% *w*/*v* of honey) was similar to those recorded by other authors [24,25].

Chestnut sample C17 (hive No. 5, CF) from 2015 showed the highest overall antimicrobial effectiveness (MIC 1.56% against *B. cereus* ATCC 11778, and MIC 6.25% against *Y. enterocolitica* ATCC 23715, *Y. enterocolitica* -food isolate, *S. aureus*—food isolate, and against *S. aureus* ATCC 25923). When we look at the phenolic composition of this sample, the main phenolic acids were ferulic, vanillic and benzoic acid, and the main flavonoids were hesperetin, pinobanksin and pinocembrin. Thus, we assume that the antibacterial activity of the sample was mediated via some or all of these compounds. Moreover, Nolan et al. [26] revealed the antimicrobial mechanism of action of ferulic acid to be dysfunction of the cell membrane and changes in the cell morphology, and that of pinocembrin to be induction of cell lysis. Matejczyk et al. [27] revealed the antibacterial activity of vanillic acid, Friedman et al. [28] evaluated the antibacterial activity of benzoic acids. Choi et al. [29] uncovered the potent antibacterial activity of hesperetin, while Darwish et al. [30] showed the antibacterial activity of pinobanksin-3-*O*-acetate and pinocembrin. The chestnut aggregation sample C16 from 2015 showed high antimicrobial effectiveness against *B. cereus* ATCC 11778 (MIC 3.13%), *Y. enterocolitica* ATCC 23715 and its food isolate and (MIC 6.25%), and *S. aureus* ATCC 25923 and its food isolate (MIC 6.25%). This sample had the same predominant phenolic acids as C17, while among the flavonoids, the predominant ones were hesperetin, chrysin and pinobanksin. Therefore, we assume that the flavonoid pattern had a significant effect on the antimicrobial activity of honey. Instead of pinocembrin, in this sample, among the three predominant flavonoids, was chrysin, whose antimicrobial mechanism of action is inhibition of DNA gyrase [26]. Based on this difference, we conclude that pinocembrin (induction of cell lysis) contributed more significantly to the antibacterial activity of honey than chrysin (inhibition of DNA gyrase). High antimicrobial activity was also shown by chestnut centrifuged sample C9v from 2014 against *B. cereus* ATCC 11778 (MIC 3.13%), *Y. enterocolitica* food isolate (MIC 6.25%), and *S. aureus* food isolate (MIC 6.25%). In this honey sample, the predominant phenolic acids were ferulic, vanillic and syringic acid, and the main flavonoids were hesperetin, pinobanksin and 3′-hydroxydaidzein. According to Nolan et al. [26], syringic acid increases cell membrane dysfunction in bacteria. The chestnut aggregation sample C8 from 2014 showed the highest antimicrobial effectiveness against *B. cereus* ATCC 11778 (MIC 3.13%), *Y. enterocolitica* food isolate (MIC 6.25%), and *S. aureus* food isolate (MIC 6.25%). Sample C8 had ferulic, protocatechuic and vanillic acid as the main phenolic acids, while the main flavonoids were pinobanksin, hesperetin and kaempferol. The antimicrobial mechanism of action of kaempferol is similar to that of chrysin, as it inhibits DNA gyrase, while for protocatechuic acid, it is still unknown [26]. The centrifuged chestnut sample C4v (main identified polyphenols: protocatechuic acid, ferulic acid, vanillic acid, hesperetin, 3′-hydroxydaidzein and pinobanksin) and the non-centrifuged sample C4 (main polyphenols: protocatechuic acid, ferulic acid, benzoic acid, hesperetin, pinobanksin and 3′-hydroxydaidzein) from 2013 had the same high (MIC 6.25%) antibacterial activity against *B. cereus* ATCC 11778.

The acacia non-centrifuged sample A9 (main identified polyphenols: chlorogenic acid, 4-coumaric acid, ferulic acid and benzoic acid, pinobanksin, pinocembrin, galangin) and the aggregation sample A8 (main identified polyphenols: chlorogenic acid, *p*-coumaric acid, benzoic acid, pinobanksin, chrysin and pinocembrin) from 2015 both showed high (MIC 3.13 and 6.25%, respectively) antibacterial activity against *B. cereus* ATCC 11778. Chlorogenic acid increases membrane permeability, resulting in cytoplasmic and nucleotide leakage, while *p*-coumaric acid disrupts the cell membrane and binds to bacterial DNA [26]. The chestnut aggregation sample C16 from 2015 (main identified polyphenols: ferulic acid, vanillic acid, benzoic acid, hesperetin, chrysin, pinobanksin) showed higher antimicrobial effectiveness against most tested pathogenic bacteria compared to the acacia aggregation sample A8 from 2015 (main identified polyphenols: chlorogenic acid, *p*-coumaric acid, benzoic acid, pinobanksin, chrysin and pinocembrin). Since peroxide (hydrogen peroxide) and non-peroxide components (flavonoids and other phenolics) are responsible for the antibacterial activity of honey [31], we propose that the antibacterial activity of our samples was also due to the synergic influence of these components.

### 3.3. Element Content by Total Reflection X-Ray Spectrometry (TXRF)

The macroelements, microelements and toxic metals were measured in the acacia and chestnut honey samples. The element levels in the acacia honey were in the following ranges: K 25.75–79.57 mg/kg, Ca 3.72–15.16 mg/kg, P 0.00–20.79 mg/kg, Cl 0.00–3.39 mg/kg, Cr 0.00–1.10 mg/kg, Mn 0.05–0.36 mg/kg, Fe 0.19–1.54 mg/kg, Ni 0.00–0.14 mg/kg, Cu 0.28–1.67 mg/kg, Zn 0.11–0.29 mg/kg, Br 0.00–3.16 mg/kg, Rb 0.00–0.20 mg/kg, and Pb 0.00–0.11 mg/kg. S and Sr were not detected in any of the acacia honey samples. These ranges are similar to or lower than the values detected by Bilandžić et al. [32]. Furthermore, same authors detected significant differences in the concentration of certain elements (Ba, Ca, Cu, Mg and Zn) between the investigated seasons (2013, 2015 and 2016) in acacia honey originating from the same geographical region as in our study (Central Croatia). Regarding the main elements in the acacia honey aggregation samples A and A1 from 2013 and the aggregation sample A8 from 2015, we recorded 1.3 and 1.6 higher values for P, 1.3 and 0.8 higher values for K, and 1.7 and 1.1 for Ca, respectively. According to Kadri et al. [16], higher values of most minerals (K, Ca, Mg, Na, Fe, Li, Zn) were measured in pressed honey compared to centrifuged honey. In our study, we observed the opposite trend for the acacia samples. The highest concentrations of K and Ca were detected in the centrifuged acacia honey sample A7 from 2013. The non-centrifuged acacia honey sample A11 from 2015 had the lowest values of P, S, Cl, K and Zn. Higher values of K and Ca were measured in the centrifuged (A7) compared to the non-centrifuged (A1-A6) acacia honey samples. Kadri et al. [16] extracted honey using a manual commercial stainless steel honey press; however, we extracted honey using natural flow. So, this could be the reason for the difference in the results of the element measurements in acacia honey. The element levels in chestnut honey were determined in the following ranges: K 283.33–846.78 mg/kg, Ca 31.66–88.72 mg/kg, P 1.36–6.63 mg/kg, Cl 1.47–8.71 mg/kg, Cr 0.0–2.15 mg/kg, Mn 3.99–10.38 mg/kg, Fe 0.33–2.35 mg/kg, Ni 0.0–0.17 mg/kg, Cu 0.37–0.86 mg/kg, Zn 0.21–0.79 mg/kg, Br 0.14–23.40 mg/kg, Rb 2.75–6.98 mg/kg, Pb 0.11–0.19 mg/kg, S 0.0–1.86 mg/kg and Sr 0.0–0.19 mg/kg. These ranges are similar to or lower than the values detected by Bilandžić et al. [32]), and higher than in our acacia samples. Significant differences in the concentration of some elements (Al, Ba, K, Mn) between some investigated seasons (2013, 2015 and 2016) were observed in chestnut honey from Central Croatia [32]. The aggregation sample C1v from 2013 and C8 from 2014 had a 1.6 times higher value for K and Ca, and 1.6 and 1.2 times higher value for Mn, compared to the aggregation sample C16 from 2015, respectively. Higher values of K and Ca were recorded in the non-centrifuged (C2, C3, C4) compared to the centrifuged (C2v, C3v, C4v) chestnut honey samples. The same trend was observed by Kadri et al. [16], and the opposite in our acacia samples. The normal centrifuged samples (C3v) had 1.7, 1.2, 1.1, 1.2, 1.3, 1.1, 1.7, 1.6 times higher values for P, Cl, K, Ca, Mn, Br, Rn and Pb than the C4v sample centrifuged through nylon, respectively. High values of heavy metals indicate potential contamination in the environment [33,34,35], while very low values or those that cannot be detected are in favor of organic farming [36]. All the honey samples showed extremely low concentrations of heavy metals and relatively high concentrations of K and Ca. The obtained values can be attributed to the organic production of plants, organic bee breeding and the ecological status of honey.

### 3.4. Pearson’s Correlation Coefficient and PCA

In both the Croatian acacia and chestnut honey samples, the TiPs correlated very strongly (r = 0.99) with the TiPAs and strongly (r = 0.72 and 0.60) with the TiFs. Accordingly, the identified phenolic acids significantly contributed to the TiP values compared to the identified flavonoids. In the acacia honey samples, the TiPAs correlated very strongly with chlorogenic acid (0.80) and strongly with gallic acid (0.77), syringic acid (0.60), *p*-coumaric acid (0.77), ferulic acid (0.73) and chrysin (0.74), and in the chestnut honey, the TiPAs correlated strongly with protocatechuic acid (0.76), benzoic acid (0.70) and syringic acid (0.73). Accordingly, the phenolic acids in the acacia honey that significantly contributed to the TiPAs were chlorogenic acid, gallic acid, 4-coumaric acid, ferulic acid and syringic acid, and in the chestnut honey, they were protocatechuic acid, syringic acid and benzoic acid. The TiFs correlated very strongly with Chl (0.87), pinobanksin (0.97), kaempferol (0.80), apigenin (0.96), pinocembrin (0.91) and galangin (0.98) and strongly with syringic acid (0.60) and chrysin (0.74) in the acacia honey and very strongly with pinocembrin (0.80) and strongly with vanillic acid (0.60), syringic acid (0.63), apigenin (0.75), chrysin (0.76) and galangin (0.76) in the chestnut honey. Accordingly, the flavonoids in the acacia honey that significantly contributed to the TiFs were galangin, pinobanksin, apigenin, pinocembrin, kaempferol and chrysin, and in the chestnut honey, they were pinocembrin, chrysin, galangin and apigenin. In both the Croatian acacia and chestnut honey types, the TPs correlated very strongly with the antioxidant activity measured by the FRAP method (r = 0.99 and 0.96). Flanjak et al. (2016) also detected very strong correlation between the phenolic content (TP) and the antioxidant activity (FRAP; 0.987). This is in correspondence with the thesis that phenolic compounds contribute the most to the antioxidant properties of honey [2,15]. In the acacia honey samples, very strong correlation was observed between P and Cr (0.92), between P and Fe (0.86), between P and Cu (0.94), between Cl and K (0.90), between Cl and Ca (0.80), between K and Rb (0.84), between Cr and Fe (0.93), between Cr and Cu (0.94) and between Fe and Cu (0.89), and in the chestnut honey samples, between S and Cr (0.84), Cr and Fe (0.91), and Mn and Rb (0.96). According to Bogdanov et al. [37], no significant correlations were observed between Pb and the majority of elements in the acacia, chestnut and other types of honey. In our acacia honey, no significant correlation was observed between Pb and all the measured elements, and in the chestnut honey, significant moderate (0.40–0.59) correlation was observed only between Pb and Rb (0.46). The PCA plots provide an overview of the similarities and differences between the different Croatian acacia honey samples stored at room temperature (A 2013 RT) after being collected in 2013 and 2015 and at the low temperature of 4 °C (A 2013 4 °C) and the Croatian chestnut honey samples stored at room temperature after being collected in 2013, 2014 and 2015 (C 2013 RT, C 2014 RT, C 2015 RT). Additionally, the interrelationships between the measured properties (phytochemical composition and antioxidant activity and measured elements) for the honey stored at RT were also visualized. The lowest distance was observed between the acacia aggregation samples A 2013 RT and A 2015 RT, and between the chestnut aggregation samples C 2013 RT and C 2014 RT, which points to the highest similarity between these samples based on the phytochemical composition and antioxidant activity. The total phenolics, individual compounds (pinobanksin, syringic acid, apigenin, chrysin and pinocembrin) and antioxidant (FRAP) activity contributed the most to the separation of the aggregation acacia honey sample A 2013 4 °C. These results indicate that the TPs and individual (pinobanksin, syringic acid, apigenin, chrysin and pinocembrin) polyphenolic compounds in the A 2013 4 °C sample are closely related to the antioxidant activity. The lowest distance, which points to the smallest difference, was detected between the C 2015 RT and C 2014 RT samples, and between A 2013 RT and A 2015 RT for the measured elements. Sample C 2013 RT had strong loadings with most elements (Cr, Sr, Rb, Mn, K, Ca, Pb) compared to samples C 2014 RT and C 2015 RT (Cl, Br, Cu, Fe, Ni and Zn) and sample A 2013 RT (P and Ga).

## 4. Materials and Methods

### 4.1. Honey Samples

Samples of acacia (9 samples produced in June 2013, and 4 samples produced in June 2015) and chestnut (10 samples produced in June 2013, 10 samples produced in June 2014, and 2 samples produced in June 2015) honey were provided by the family farm “OPG Davorka Franić” from Klinac (45°22′29.6′′ N and 16°18′27.5′′ E), Banija, in Sisačko-Moslavačka county, Croatia. Samples of honey were collected from different hive positions (on the edge 1–3 or 42–44, and in the middle of a series of hives) and parts of the hive (small or normal part), and two different honey extraction methods were used (centrifuging or draining honey combs). The honey samples were characterized by the Croatian Regulations [38] and harmonized methods of the European Commission [39] (Table S4). The samples were stored for two months at room temperature (RT) in a dark place, and at low temperature (4 °C) in a refrigerator, until purification. Names and descriptions of the honey samples are listed in Table S5.

### 4.2. Chemicals and Apparatus

Phenolic acids, flavonol glycosides and aglycones were purchased from Sigma-Aldrich GmbH (Steinheim, Germany) and Extrasynthese (Genay, France). All the chemicals and reagents were of analytical grade and supplied by Sigma Aldrich GmbH (Taufkirchen, Germany) or Kemika (Zagreb, Croatia). The RP-HPLC analyses were performed using the Agilent 1100 Series system equipped with a quaternary pump, multiwave UV/Vis detector, autosampler, fraction collector, analytical Zorbax Rx-C18 guard column (4.6 × 12.5 mm, 5 µm particle size) and Poroshell 120 SB-C18 column (4.6 × 75 mm, 2.7 µm particle size) (Agilent Technologies, Waldbronn, Germany). The absorbance measurements were performed with a Fluostar Optima microplate reader (BMG Labtech GmbH, Offenburg, Germany). The mineral measurements were performed on a TXRF spectrometer (S2 PICOFOX, Bruker AXS Microanalysis GmbH, Berlin, Germany).

### 4.3. Extraction of Phenolics Compounds from Honey Samples

The phenolic acid and flavonoid extractions from the honey samples were performed as described in Kenjerić et al. [13] with slight modification. A sample of 25 g honey was dissolved in 200 mL of distilled water acidified with hydrochloric acid (pH 2) and stirred for 30 min on a magnetic stirrer. The solution was filtered through cotton wool to remove solid particles and then slowly passed through a glass column (42 cm × 3.2 cm) filled with 50 g of previously washed (with 200 mL of methanol and 200 mL of distilled water) Amberlite XAD-2 resin (Supelco, Bellefonte, PA, USA, pore size of 9 nm, particle size 0.3 to 1.2 mm). The resins were washed with 250 mL of acidified distilled water (pH 2), followed by 250 mL of distilled water, to rinse out all the sugars and other polar components of honey. The phenolic compounds were extracted with 200 mL of methanol (HPLC grade). The methanol extract was concentrated under a vacuum at 40 °C and suspended in 10 mL of distilled water. The sample was extracted three times with 5 mL of ethyl–acetate. All three ethyl–acetate fractions were combined and the sample was dried under a vacuum at 40 °C. Thus, the prepared ethyl–acetate extract was diluted in 96% ethanol and stored in a freezer at −20 °C until further analysis.

### 4.4. Spectrophotometric Determination of Total Polyphenols and Antioxidant Activity

The total polyphenols (TPs) were determined with Folin–Ciocalteu reagent according to Zhishen et al. [40]. A volume of 2 μL of tested solution was diluted with 158 μL of deionized water and then 10 μL of Folin–Ciocalteu reagent was added. Afterwards, 30 μL Na_2_CO_3_ (1.88 M) was added, and the mixture was incubated for 30 min at 45 °C. The absorbance of the mixture was measured at 740 nm. The TPs content was calculated from the calibration curve and expressed as gallic acid equivalents (GAEs). The ferric-reducing antioxidant power (FRAP) assay was performed according to the original method of Benzie and Strain [41]. The tested solution (10 μL) was mixed with the freshly prepared FRAP reagent (190 μL) and the absorbance was read at 595 nm after 4 min of reaction time. A calibration curve was constructed for FeSO_4_ × 7H_2_O and the results were expressed as μM Fe ^2+^.

### 4.5. RP-HPLC Analysis of Phenolic Acids and Flavonoids

Qualitative and quantitative reversed-phase high-performance liquid chromatography (RP-HPLC) analyses of the honey extracts were performed using the Agilent 1100 Series system as described in Vujčić Bok et al. [42]. The solvents used were as follows: (A) 0.2% (*v*/*v*) aqueous glacial acetic acid, and (B) 80% (*v*/*v*) methanol + 0.2% (*v*/*v*) glacial acetic acid. The gradient profile was as follows (A/B): 85/15 at 0 min, 51.7/48.3 at 20 min, 46.5/53.5 at 24 min, 36.5/63.5 at 30 min, 0/100 at 37.3 min, 0/100 at 40 min, 100/0 at 43 min. The injection volume was 10 µL, the constant flow rate was 1.0 mL/min, and the column temperature was set at 30 °C. The multiwave UV/Vis detector was set at 280 nm. The phenolic compounds were characterized according to their retention times and the UV spectra were compared with commercial standards. For the quantitative analyses, calibration curves were obtained by injection of 8 known concentrations (in the range 1–250 µg/mL) of the mixed 96% EtOH standard solution in triplicate. The injection volume was 5 µL. The honey extracts were compared with the available phenolic standards. The calibration curves were made by plotting the mean peak area versus the concentration of the standards, and they are shown together with their *R*^2^ values in Table S6. The phenolics quantification was made by integration of the peak areas with reference to the calibration curves made using known amounts of the available pure standard compounds. The results were expressed as mg/kg of honey weight ± SD.

### 4.6. Total Reflection X-Ray Fluorescence (TXRF)

The collected samples were packed and stored below 20 °C prior to analysis. Approximately 0.25 g of honey samples was weighed into the 3 mL plastic tube and mixed with 1 mL of ultrapure water. The solution was shaken until all of the honey was dissolved, and then the solution was homogenized in an ultrasonic bath for approximately 10 min. The sample solutions were prepared by weighing 450 µL of each honey sample solution and adding 50 µL of Ga standard solution, and then thoroughly homogenized, to prepare the specimen for TXRF analysis with a final Ga concentration of 1 mg/L. A volume of 10 µL of the obtained solution was deposited into a silicon quartz reflector and dried on a heating plate at temperatures of approximately 50 °C. The sample, as a very thin dry honey residue on the reflector, was then measured by TXRF. From each specimen solution, three replicates were analyzed. All the measurements were performed on a benchtop TXRF spectrometer. The instrument was equipped with an Mo target microfocus tube, a multilayer monochromator and a 30 mm^2^ XFlash silicon drift detector. The operating conditions were 50 kV and 750 µA and the acquisition time for all the spectra was set to 600 s (measurement time). The measurements were performed in air. Quantitative analysis based on the internal standard of a known concentration (Ga), which is not present in the sample, was straightforward because the sample was thin, and the matrix corrections could be neglected.

### 4.7. Antibacterial Activity

Investigation of the antibacterial activity of the ten selected honey samples was carried out in the Croatian Veterinary Institute, Zagreb, by the microdilution method, measuring the minimal inhibitory concentration (MIC) values in microtiter plates (96 wells) against different reference (ATCC) and wild-type (WT) bacterial strains. The selection of microorganisms was based on their incidence as causative agents of human food-borne diseases, as well as with the purpose to perceive differences, if they exist, in the effects between the antimicrobial activity of different honey samples on the reference strains compared to their ‘wild types’ that the population frequently encounters. The selected Gram-positive reference strains included in the study were *Bacillus cereus* ATCC 11778, *Staphylococcus aureus* ATCC 25923, and *Listeria monocytogenes* ATCC 13932, while the Gram-negative reference strains were *Escherichia coli* ATCC 25922, *Salmonella* ser. Enteritidis ATCC 13076, *Pseudomonas aeruginosa* ATCC 27583, and *Yersinia enterocolitica* ATCC 23715. The wild-type bacterial strains of the same bacterial species were in–house and obtained from routine food analysis. Suspensions of the microorganisms were prepared from a pure overnight culture grown on a nutrient agar (Merck, Darmstadt, Germany), after which several colonies were suspended in 3 mL of sterile saline (Saline solution, bioMerieux, Marcy l’Etoile, France) and adjusted to McFarland 0.5 (VITEK Densichek, bioMerieux, Marcy l’Etoile, France). Out of the thus obtained suspension for each particular microorganism, a 50 µl of 0.5 McFarland was added into 10 mL of Mueller–Hinton broth to gain a final solution of about 10^6^ CFU/mL. For the purity control check, 10 µL of bacterial suspension was spread on nutrient agar and incubated at 37 °C for 24 h. Stock solutions were prepared to obtain the primary solution of 50% w/v by adding and mixing 2 g of honey to 2 mL of sterile saline water, and serial dilutions of the stock solutions of 25% *w*/*v*, 12.5% *w*/*v*, 6.25% *w*/*v*, 3.13% *w*/*v*, 1.56% *w*/*v*, 0.78% *w*/*v*, 0.39% *w*/*v*, 0.19% *w*/*v*, 0.09% *w*/*v*. Microtiter trays (96 wells) with the prepared serial dilutions of the samples were then inoculated with 50 µL of the inoculum of microorganism suspension using a multi-channel pipette, sealed and incubated at 37 °C for 18–22 h. For a sterility check, 100 µL of Mueller–Hinton broth, and 50 µL of Mueller–Hinton broth mixed with 50 µL of stock solution for a growth check, respectively, have been added into the wells of the microtiter tray columns before those with the dilutions of the samples. Each sample was tested in triplicate. The minimum inhibitory concentration (MIC) was defined as the smallest percentage solution able to inhibit the growth of the tested microorganisms.

### 4.8. Statistical Analysis

All the results were evaluated using the Statistica 13.3 software package (Stat Soft Inc., Tulsa, OK, USA). The RP-HPLC and results from the spectrophotometric measurements were subjected to one-way ANOVA for comparison of the means and the significant differences were calculated according to Duncan’s multiple range test. The data are presented as the mean ± standard deviation (SD). The Pearson’s correlation coefficient and principal component analysis (PCA) between the phytochemicals were performed. Data were considered statistically significant at *p* ≤ 0.05.

## 5. Conclusions

Croatian acacia and chestnut honeys’ polyphenolic and elemental composition, as well as the antioxidant and antimicrobial activity, were analyzed from several aspects: production season, hive position (on the edge and in the middle of a series of hives), part of the hive (small or normal extension), and extraction method (centrifuging or draining honey combs). In the samples of acacia honey, we also monitored the influence of different storage temperatures (room temperature and 4 °C). According to the results, storage at room temperature was better for the stability of the phenolic acids in acacia honey. The flavonoids in the acacia honey samples were less affected by the storage conditions (temperature). In 2013, higher values for the polyphenols and antioxidant capacity were observed in the non-centrifuged honey samples from the small extension of the hive and collected in the middle of a series of hives. The sample that was centrifuged through nylon had significantly lower values of total phenolics and antioxidant capacity (FRAP) than the normally centrifuged honey sample. Regarding the antibacterial activity, the centrifuged chestnut honey collected from the edge of the hives in the year 2015 showed the highest overall effectiveness. All the honey samples showed extremely low concentrations of heavy metals and relatively high concentrations of potassium and calcium. Principal component analysis showed that both the phenolic composition and the elemental profile might be used for the clear separation of acacia and chestnut honey. This shows that the phenolic composition and the elemental profile are unique enough to differentiate between acacia honey and chestnut honey. Furthermore, honey stored at room temperature could clearly be separated from that stored at 4 °C, which suggests that the storage temperature influences the chemical stability, degradation, or transformation of honey compounds over time. The results highlight PCA’s utility in honey authentication, quality control, and storage studies. We conclude that the phenolic and elemental profiles can be used as markers for honey authentication, helping to confirm the botanical origin (acacia vs. chestnut). The hive position, part of the hive, extraction method and storage conditions should be carefully managed to preserve honey’s original chemical characteristics, which are critical for its quality, health benefits and regulatory standards. Through being able to screen these parameters in time, the proposed analyses might help producers to segregate samples of the highest quality from each of the honey types.

## Figures and Tables

**Table 1:** The most common phenolic acids in the acacia honey samples are presented in bold. Values are the mean ± SD based on three replicates. Different letters in a column are significantly different at *p* < 0.05. s = small extension = half-super (part of the hive), * = repeated sample triplicate.

	*p*-Coumaric Acid				Gallic Acid				Chlorogenic Acid				Ferulic Acid				Benzoic Acid				Syringic Acid			
A	**31.42**	**±**	**0.07**	**a**	**83.01**	**±**	**1.70**	**a**	18.86	±	0.23	c	**24.38**	**±**	**0.43**	**a**	6.29	±	0.09	c	2.37	±	0.20	cbd
A1	**3.78**	**±**	**0.02**	**r**	**22.00**	**±**	**3.60**	**e**	3.07	±	0.15	lm	1.98	±	0.06	n	2.65	±	0.17	hi	**4.91**	**±**	**2.80**	**cb**
A10	**5.56**	**±**	**0.12**	**n**	0.50	±	0.03	h	**4.30**	**±**	**0.06**	**ij**	**3.60**	**±**	**0.07**	**l**	1.84	±	0.01	l	0.00	±	0.00	d
A11	**2.98**	**±**	**0.18**	**s**	0.61	±	0.04	h	**2.69**	**±**	**0.11**	**mn**	**1.93**	**±**	**0.09**	**n**	1.47	±	0.06	m	0.00	±	0.00	d
A1f	**6.52**	**±**	**0.23**	**m**	3.24	±	0.53	gh	**4.64**	**±**	**0.20**	**i**	4.41	±	0.11	j	3.71	±	0.15	e	3.90	±	0.04	cb
A1f*	**4.89**	**±**	**0.18**	**q**	3.21	±	0.64	gh	**4.36**	**±**	**0.13**	**i**	**3.34**	**±**	**0.07**	**l**	**4.38**	**±**	**0.06**	**d**	3.04	±	0.18	cbd
A2	**25.84**	**±**	**0.12**	**b**	**22.27**	**±**	**3.12**	**e**	**21.97**	**±**	**0.41**	**a**	17.62	±	0.25	b	2.33	±	0.18	jk	5.58	±	3.54	b
A2f	**23.61**	**±**	**0.43**	**c**	2.89	±	0.26	gh	14.91	±	0.26	e	**17.88**	**±**	**0.53**	**b**	2.27	±	0.04	jk	0.00	±	0.00	d
A3	**9.58**	**±**	**0.07**	**i**	**13.90**	**±**	**2.04**	**f**	**5.64**	**±**	**0.11**	**h**	**6.46**	**±**	**0.05**	**g**	1.15	±	0.06	n	0.00	±	0.00	d
A3f	**8.81**	**±**	**0.05**	**j**	2.97	±	0.41	gh	3.77	±	0.06	jk	**6.56**	**±**	**0.12**	**g**	1.72	±	0.07	lm	0.00	±	0.00	d
A4	10.71	±	0.15	h	6.16	±	0.56	g	**18.06**	**±**	**0.48**	**d**	5.77	±	0.20	h	**18.47**	**±**	**0.38**	**a**	0.00	±	0.00	d
A4f	**13.73**	**±**	**0.17**	**f**	1.54	±	0.30	h	10.49	±	0.14	f	9.77	±	0.14	d	**9.95**	**±**	**0.18**	**b**	3.64	±	0.14	cbd
A4s	**11.36**	**±**	**0.16**	**g**	**55.55**	**±**	**3.55**	**b**	**7.87**	**±**	**0.18**	**g**	8.82	±	0.41	e	2.31	±	0.11	jk	4.08	±	1.98	cb
A4s*	**18.06**	**±**	**0.73**	**d**	**39.29**	**±**	**2.90**	**c**	10.64	±	0.94	f	**12.06**	**±**	**0.47**	**c**	3.28	±	0.11	fg	2.79	±	1.85	cbd
A5	**16.16**	**±**	**0.47**	**e**	**34.12**	**±**	**2.97**	**d**	10.73	±	0.38	f	7.96	±	0.16	f	3.33	±	0.27	fg	**3.58**	**±**	**2.03**	**cbd**
A5f	**8.38**	**±**	**0.20**	**k**	1.86	±	0.40	h	**4.59**	**±**	**0.13**	**i**	**5.21**	**±**	**0.19**	**i**	2.22	±	0.08	jk	1.70	±	0.09	cd
A6	9.59	±	0.30	i	20.70	±	2.66	e	**19.62**	**±**	**0.73**	**b**	6.63	±	0.12	g	4.18	±	0.33	d	17.51	±	7.35	a
A6f	**4.94**	**±**	**0.10**	**q**	2.60	±	0.32	h	7.68	±	0.19	g	**3.99**	**±**	**0.09**	**k**	2.71	±	0.09	h	0.00	±	0.00	d
A7	**4.46**	**±**	**0.01**	**r**	**15.90**	**±**	**1.73**	**f**	**2.47**	**±**	**0.03**	**n**	**2.96**	**±**	**0.02**	**m**	1.21	±	0.07	n	1.97	±	1.27	cbd
A7f	**7.46**	**±**	**0.30**	**l**	3.51	±	0.64	gh	4.45	±	0.10	i	**5.39**	**±**	**0.10**	**i**	2.45	±	0.04	ij	0.00	±	0.00	d
A7f*	**6.93**	**±**	**0.19**	**m**	3.37	±	0.37	gh	3.72	±	0.19	k	**5.12**	**±**	**0.11**	**i**	2.13	±	0.08	k	**5.02**	**±**	**0.35**	**cb**
A8	**3.29**	**±**	**0.14**	**s**	1.51	±	0.08	h	**3.40**	**±**	**0.05**	**kl**	2.17	±	0.06	n	**3.15**	**±**	**0.04**	**g**	0.00	±	0.00	d
A9	**5.64**	**±**	**0.23**	**n**	0.97	±	0.11	h	**8.03**	**±**	**0.24**	**g**	**3.42**	**±**	**0.14**	**l**	**3.45**	**±**	**0.06**	**f**	0.00	±	0.00	d

**Table 2:** The most common flavonoids in the acacia honey samples are presented in bold. Values are the mean ± SD based on three replicates. Different letters in a column are significantly different at *p* < 0.05. s = small extension = half-super (part of the hive), * = repeated sample triplicate.

	Pinobanksin				Pinocembrin				Galangin				Chrysin				Apigenin				Kaempferol				Quercetin			
A	**3.30**	**±**	**0.02**	**f**	**2.17**	**±**	**0.01**	**e**	0.89	±	0.06	g	**1.27**	**±**	**0.02**	**g**	**1.79**	**±**	**0.04**	**b**	0.58	±	0.10	a	0.08	±	0.00	i
A1	**1.33**	**±**	**0.04**	**j**	**0.54**	**±**	**0.02**	**mn**	0.28	±	0.04	l	**0.91**	**±**	**0.02**	**hi**	0.47	±	0.06	jk	0.09	±	0.01	l	0.28	±	0.03	fg
A10	**1.45**	**±**	**0.05**	**j**	**1.00**	**±**	**0.05**	**k**	**0.59**	**±**	**0.04**	**hi**	0.47	±	0.01	l	**0.59**	**±**	**0.03**	**hij**	0.24	±	0.01	efg	0.17	±	0.02	h
A11	**0.58**	**±**	**0.03**	**l**	0.28	±	0.01	o	0.11	±	0.01	m	0.24	±	0.02	n	**0.38**	**±**	**0.06**	**klm**	0.21	±	0.01	fghij	**0.31**	**±**	**0.01**	**def**
A1f	**2.81**	**±**	**0.08**	**g**	**1.09**	**±**	**0.02**	**j**	0.46	±	0.04	j	**1.60**	**±**	**0.07**	**f**	0.83	±	0.06	efg	0.15	±	0.01	ijkl	0.41	±	0.03	ab
A1f*	**3.48**	**±**	**0.11**	**f**	**1.28**	**±**	**0.04**	**i**	0.68	±	0.04	h	**1.99**	**±**	**0.08**	**d**	1.00	±	0.07	e	0.17	±	0.02	ghijk	0.35	±	0.02	cd
A2	**8.64**	**±**	**0.05**	**b**	2.44	±	0.02	d	**2.68**	**±**	**0.09**	**b**	**2.59**	**±**	**0.02**	**b**	2.21	±	0.22	a	0.65	±	0.06	a	0.19	±	0.03	h
A2f	**5.19**	**±**	**0.22**	**d**	**3.25**	**±**	**0.11**	**b**	**2.33**	**±**	**0.19**	**d**	1.93	±	0.27	d	1.56	±	0.32	c	0.61	±	0.08	a	0.27	±	0.01	fg
A3	**0.45**	**±**	**0.02**	**l**	0.14	±	0.00	q	0.05	±	0.00	m	**0.26**	**±**	**0.00**	**n**	**0.26**	**±**	**0.03**	**lm**	**0.49**	**±**	**0.02**	**b**	0.19	±	0.01	h
A3f	**0.56**	**±**	**0.04**	**l**	0.21	±	0.00	p	0.05	±	0.00	m	**0.44**	**±**	**0.01**	**lm**	0.25	±	0.03	lm	0.11	±	0.02	k	**0.34**	**±**	**0.03**	**cde**
A4	**1.44**	**±**	**0.04**	**j**	0.29	±	0.01	o	0.24	±	0.02	l	**0.48**	**±**	**0.02**	**l**	**0.70**	**±**	**0.02**	**fh**	0.26	±	0.06	def	0.08	±	0.00	i
A4f	**1.48**	**±**	**0.02**	**j**	**0.98**	**±**	**0.03**	**k**	0.35	±	0.05	kl	**0.42**	**±**	**0.02**	**lm**	**0.66**	**±**	**0.05**	**ghi**	0.41	±	0.01	c	0.08	±	0.00	i
A4s	**1.14**	**±**	**0.09**	**jk**	**1.73**	**±**	**0.06**	**g**	0.90	±	0.01	g	**3.10**	**±**	**0.07**	**a**	0.42	±	0.14	jkl	0.27	±	0.03	def	0.45	±	0.04	a
A4s*	2.50	±	0.12	h	0.68	±	0.02	l	0.44	±	0.02	jk	0.78	±	0.01	ij	0.53	±	0.11	hijk	0.29	±	0.06	de	0.45	±	0.11	a
A5	**3.82**	**±**	**0.19**	**e**	**1.35**	**±**	**0.04**	**h**	1.17	±	0.07	f	**2.29**	**±**	**0.09**	**c**	0.88	±	0.07	ef	0.23	±	0.02	efgh	0.08	±	0.00	i
A5f	**1.78**	**±**	**0.02**	**i**	**1.72**	**±**	**0.04**	**g**	1.11	±	0.09	f	**2.36**	**±**	**0.11**	**c**	0.41	±	0.06	jklm	0.14	±	0.02	jkl	0.29	±	0.03	efg
A6	**12.96**	**±**	**0.21**	**a**	**3.07**	**±**	**0.05**	**c**	**3.03**	**±**	**0.15**	**a**	**2.67**	±	0.07	b	2.30	±	0.11	a	0.47	±	0.03	bc	0.08	±	0.00	i
A6f	**5.23**	**±**	**0.17**	**d**	**2.11**	**±**	**0.02**	**f**	**1.79**	**±**	**0.07**	**e**	**1.80**	**±**	**0.04**	**e**	1.24	±	0.12	d	0.32	±	0.02	d	0.08	±	0.00	i
A7	**0.59**	**±**	**0.01**	**l**	**0.23**	**±**	**0.00**	**op**	0.10	±	0.01	m	**0.32**	**±**	**0.01**	**mn**	**0.22**	**±**	**0.02**	**m**	0.09	±	0.01	l	0.25	±	0.00	g
A7f	**1.36**	**±**	**0.07**	**j**	**0.52**	**±**	**0.01**	**n**	0.31	±	0.02	l	**0.63**	**±**	**0.04**	**k**	0.48	±	0.05	jk	0.18	±	0.02	ghijk	0.37	±	0.04	bc
A7f*	**1.16**	**±**	**0.05**	**jk**	**0.67**	**±**	**0.02**	**l**	0.31	±	0.01	l	**1.04**	**±**	**0.03**	**h**	0.37	±	0.05	klm	0.16	±	0.03	hijk	0.35	±	0.03	cd
A8	**1.33**	**±**	**0.06**	**jk**	**0.59**	**±**	**0.02**	**m**	0.49	±	0.02	ij	**0.73**	**±**	**0.03**	**jk**	0.40	±	0.03	klm	0.21	±	0.01	fghi	0.08	±	0.00	i
A9	**6.77**	**±**	**0.22**	**c**	**3.62**	**±**	**0.05**	**a**	**2.55**	**±**	**0.09**	**c**	**1.49**	**±**	**0.17**	**f**	1.80	±	0.08	b	0.47	±	0.03	bc	0.08	±	0.00	i

**Table 3:** The most common phenolic acids in the chestnut honey samples are presented in bold. Values are the mean ± SD based on three replicates. Different letters in a column are significantly different at *p* < 0.05. s = small extension = half-super (part of the hive).

	Protocatechuic Acid				Vanillic Acid				Ferulic Acid				Benzoic Acid				Syringic Acid			
C10	**5.36**	**±**	**0.85**	**de**	1.30	±	0.02	j	**10.37**	**±**	**0.10**	**d**	0.76	±	0.08	p	**2.90**	**±**	**0.07**	**e**
C10N	**5.04**	**±**	**0.41**	**e**	**4.46**	**±**	**0.02**	**d**	**6.99**	**±**	**0.09**	**j**	1.63	±	0.13	hi	3.39	±	0.12	d
C11	**7.76**	**±**	**0.85**	**c**	1.02	±	0.01	l	**5.39**	**±**	**0.02**	**n**	**1.82**	**±**	**0.03**	**fg**	0.83	±	0.18	k
C12	**7.57**	**±**	**1.01**	**c**	1.16	±	0.02	k	**8.49**	**±**	**0.13**	**g**	**1.62**	**±**	**0.05**	**hi**	1.59	±	0.07	h
C13	**1.96**	**±**	**0.04**	**g**	1.19	±	0.01	k	**10.74**	**±**	**0.14**	**c**	1.02	±	0.05	mno	**1.58**	**±**	**0.19**	**h**
C14	**2.54**	**±**	**0.11**	**g**	1.41	±	0.02	i	**3.57**	**±**	**0.05**	**pq**	**1.68**	**±**	**0.04**	**gh**	0.81	±	0.08	k
C15	**3.63**	**±**	**0.63**	**f**	**1.94**	**±**	**0.01**	**g**	**5.81**	**±**	**0.08**	**m**	1.37	±	0.13	jk	1.03	±	0.12	ij
C16	0.60	±	0.01	h	**2.25**	**±**	**0.06**	**f**	**3.46**	**±**	**0.07**	**q**	**1.91**	**±**	**0.11**	**ef**	0.88	±	0.14	jk
C17	0.35	±	0.01	h	**2.38**	**±**	**0.07**	**e**	**4.34**	**±**	**0.05**	**o**	**2.02**	**±**	**0.08**	**e**	1.58	±	0.07	h
C1v	**3.31**	**±**	**0.03**	**f**	**1.19**	**±**	**0.03**	**k**	**4.40**	**±**	**0.03**	**o**	0.88	±	0.06	op	0.74	±	0.02	k
C2	**5.82**	**±**	**0.10**	**d**	**1.06**	**±**	**0.01**	**l**	4.42	±	0.02	o	**1.94**	**±**	**0.07**	**ef**	0.47	±	0.02	l
C2v	**2.30**	**±**	**0.02**	**g**	**1.27**	**±**	**0.04**	**j**	**5.99**	**±**	**0.09**	**l**	0.97	±	0.09	no	0.41	±	0.03	l
C3	**3.83**	**±**	**0.06**	**f**	1.28	±	0.03	j	**14.32**	**±**	**0.17**	**a**	1.36	±	0.05	jk	**2.20**	**±**	**0.02**	**f**
C3v	**3.73**	**±**	**0.08**	**f**	**1.29**	**±**	**0.04**	**j**	**6.18**	**±**	**0.01**	**k**	1.05	±	0.07	mn	1.01	±	0.03	j
C4	**30.83**	**±**	**0.21**	**a**	1.29	±	0.04	j	**7.72**	**±**	**0.09**	**h**	**2.94**	**±**	**0.01**	**c**	1.79	±	0.07	g
C4v	**5.90**	**±**	**0.10**	**d**	**1.30**	**±**	**0.01**	**j**	**3.65**	**±**	**0.02**	**p**	1.26	±	0.00	kl	1.03	±	0.02	ij
C5	4.74	±	0.19	e	**8.96**	**±**	**0.08**	**a**	**11.53**	**±**	**0.22**	**b**	3.49	±	0.26	a	**9.27**	**±**	**0.02**	**a**
C6	**13.20**	**±**	**0.10**	**b**	5.69	±	0.02	b	**9.70**	**±**	**0.03**	**e**	2.46	±	0.07	d	**6.66**	**±**	**0.05**	**b**
C7	3.54	±	0.16	f	**5.07**	**±**	**0.03**	**c**	**8.52**	**±**	**0.02**	**g**	3.15	±	0.08	b	**4.43**	**±**	**0.09**	**c**
C8	**3.34**	**±**	**0.05**	**f**	**1.61**	**±**	**0.03**	**h**	**5.47**	**±**	**0.12**	**n**	1.49	±	0.05	ij	1.17	±	0.12	i
C9	1.00	±	0.02	h	**1.96**	**±**	**0.02**	**g**	**9.11**	**±**	**0.06**	**f**	1.14	±	0.03	lm	**2.15**	**±**	**0.06**	**f**
C9v	1.00	±	0.02	h	**1.92**	**±**	**0.06**	**g**	**7.51**	**±**	**0.21**	**i**	1.04	±	0.04	mno	**1.64**	**±**	**0.07**	**gh**

**Table 4:** The most common flavonoids in the chestnut honey samples are presented in bold. Values are the mean ± SD based on three replicates. Different letters in a column are significantly different at *p* < 0.05. s = small extension = half-super (part of the hive).

	Pinobanksin				Pinocembrin				Hesperetin				Chrysin				3′-Hydroxydaidzein				Apigenin				Kaempferol				Quercetin			
C10	**2.06**	**±**	**0.02**	**a**	**0.88**	**±**	**0.02**	**b**	**1.46**	**±**	**0.04**	**ij**	0.78	±	0.01	a	0.05	±	0.01	n	0.39	±	0.15	b	0.18	±	0.01	bcd	0.26	±	0.01	c
C10N	**1.97**	**±**	**0.03**	**b**	**0.30**	**±**	**0.03**	**g**	**1.84**	**±**	**0.09**	**h**	0.25	±	0.01	e	0.17	±	0.01	jk	0.16	±	0.09	def	0.15	±	0.04	d	0.12	±	0.01	fg
C11	**0.51**	**±**	**0.03**	**g**	0.13	±	0.00	hij	**1.96**	**±**	**0.05**	**gh**	0.23	±	0.00	f	0.18	±	0.04	jk	0.18	±	0.07	de	0.16	±	0.04	cd	**0.44**	**±**	**0.06**	**a**
C12	**0.87**	**±**	**0.02**	**d**	0.58	±	0.01	d	**1.89**	**±**	**0.04**	**h**	**0.74**	**±**	**0.02**	**b**	**0.20**	**±**	**0.03**	**ijk**	0.20	±	0.06	cd	0.16	±	0.05	cd	0.38	±	0.02	b
C13	**0.60**	**±**	**0.04**	**f**	0.17	±	0.01	hi	**1.06**	**±**	**0.05**	**l**	0.07	±	0.00	l	0.29	±	0.07	h	0.09	±	0.02	efg	0.16	±	0.00	cd	0.11	±	0.03	fg
C14	**0.33**	**±**	**0.00**	**i**	0.09	±	0.01	jk	**1.33**	**±**	**0.14**	**k**	0.05	±	0.00	m	**0.25**	**±**	**0.00**	**hi**	0.18	±	0.01	de	0.18	±	0.01	bcd	0.19	±	0.04	d
C15	**0.31**	**±**	**0.02**	**i**	0.08	±	0.02	k	**2.79**	**±**	**0.08**	**d**	0.12	±	0.00	i	0.22	±	0.00	ij	0.07	±	0.01	fg	0.16	±	0.04	cd	**0.47**	**±**	**0.05**	**a**
C16	**0.60**	**±**	**0.02**	**f**	0.36	±	0.01	f	**2.52**	**±**	**0.02**	**ef**	**0.73**	**±**	**0.00**	**b**	0.07	±	0.01	mn	0.06	±	0.02	fg	0.16	±	0.06	cd	0.18	±	0.00	de
C17	**1.14**	**±**	**0.05**	**c**	**0.52**	**±**	**0.01**	**e**	**2.90**	**±**	**0.03**	**cd**	0.52	±	0.00	c	0.15	±	0.01	kl	0.28	±	0.13	c	0.20	±	0.05	bcd	0.08	±	0.00	g
C1v	**0.18**	**±**	**0.00**	**k**	0.02	±	0.00	m	**2.61**	**±**	**0.08**	**e**	0.12	±	0.00	i	0.17	±	0.01	jk	0.11	±	0.01	defg	**0.27**	**±**	**0.00**	**a**	0.08	±	0.00	g
C2	0.15	±	0.02	k	0.07	±	0.01	kl	**3.42**	**±**	**0.06**	**a**	0.09	±	0.00	k	**0.44**	**±**	**0.02**	**g**	0.18	±	0.01	de	**0.26**	**±**	**0.01**	**a**	0.08	±	0.00	g
C2v	0.10	±	0.01	l	0.06	±	0.01	klm	**2.05**	**±**	**0.02**	**g**	0.03	±	0.00	n	**1.08**	**±**	**0.02**	**c**	**0.28**	**±**	**0.02**	**cd**	0.22	±	0.00	b	0.08	±	0.00	g
C3	**0.34**	**±**	**0.02**	**i**	0.05	±	0.01	klm	**3.02**	**±**	**0.08**	**bc**	0.21	±	0.00	g	**0.91**	**±**	**0.06**	**d**	0.11	±	0.03	defg	0.30	±	0.01	a	0.08	±	0.00	g
C3v	0.10	±	0.01	l	0.03	±	0.00	lm	**1.07**	**±**	**0.03**	**l**	0.05	±	0.00	m	**0.86**	**±**	**0.05**	**e**	0.18	±	0.01	de	**0.18**	**±**	**0.01**	**bcd**	0.08	±	0.00	g
C4	**0.69**	**±**	**0.00**	**e**	0.16	±	0.01	hi	**2.88**	**±**	**0.07**	**d**	0.31	±	0.00	d	**0.44**	**±**	**0.02**	**g**	0.08	±	0.01	fg	0.26	±	0.02	a	0.08	±	0.00	g
C4v	**0.24**	**±**	**0.02**	**j**	0.09	±	0.00	jk	**1.52**	**±**	**0.04**	**i**	0.15	±	0.00	h	**0.51**	**±**	**0.03**	**f**	0.13	±	0.02	defg	0.16	±	0.01	cd	0.08	±	0.00	g
C5	1.16	±	0.02	c	**2.20**	**±**	**0.10**	**a**	**2.44**	**±**	**0.15**	**f**	0.78	±	0.01	a	**1.29**	**±**	**0.03**	**b**	0.97	±	0.01	a	0.05	±	0.00	e	0.08	±	0.00	g
C6	**0.62**	**±**	**0.04**	**f**	0.17	±	0.01	h	**1.87**	**±**	**0.06**	**h**	0.24	±	0.01	ef	**0.24**	**±**	**0.04**	**hi**	0.04	±	0.00	g	0.19	±	0.02	bcd	0.08	±	0.00	g
C7	**0.41**	**±**	**0.00**	**h**	0.14	±	0.01	hij	**3.05**	**±**	**0.07**	**b**	0.10	±	0.00	j	**1.44**	**±**	**0.02**	**a**	0.18	±	0.06	de	0.29	±	0.00	a	0.08	±	0.00	g
C8	**0.87**	**±**	**0.05**	**d**	0.16	±	0.00	hi	**0.45**	**±**	**0.09**	**m**	0.09	±	0.01	jk	0.11	±	0.02	lm	0.14	±	0.01	defg	**0.20**	**±**	**0.02**	**bc**	0.14	±	0.02	ef
C9	**0.63**	**±**	**0.00**	**f**	**0.66**	**±**	**0.01**	**c**	**1.47**	**±**	**0.07**	**ij**	0.21	±	0.01	g	0.42	±	0.01	g	0.19	±	0.01	cde	0.20	±	0.00	bc	0.16	±	0.01	de
C9v	**0.37**	**±**	**0.01**	**i**	0.13	±	0.00	ij	1.38	±	0.10	ijk	0.04	±	0.01	m	**0.28**	**±**	**0.02**	**h**	0.11	±	0.01	defg	0.16	±	0.01	cd	0.20	±	0.04	d

**Table 5:** Total phenolic content determined by the Folin–Ciocalteu method and antioxidant activity determined with the FRAP method of the Croatian acacia honey stored at room temperature (A1–A7 and A8–A11) after collection in 2013 and 2015 and at the low temperature of 4° C (A1f–A7f) after collection in 2013 and of the chestnut honey (C1–C17) samples collected in 2013, 2014 and 2015. Values are the mean ± SD based on three replicates. The statistics were determined separately for the acacia honey samples and separately for the chestnut honey samples. Different letters are significantly different at *p* < 0.05. N = normal extension (part of the hive), s = small extension = half-super (part of the hive), v = centrifuged.

Sample	TP (mg GAE/g DW)			FRAP (μM Fe(II)/g DW)				
A1	129.71	±	0.20	^p^	1487.34	±	7.08	^q^
A2	313.02	±	1.44	^k^	2626.84	±	23.63	^m^
A3	293.54	±	2.11	^l^	2338.98	±	17.28	^o^
A4	144.49	±	0.51	^o^	1644.99	±	13.96	^p^
A4s	320.80	±	2.29	^k^	2728.48	±	1.09	^l^
A5	3339.87	±	20.22	^a^	27,642.13	±	41.05	^a^
A6	649.52	±	3.94	^g^	4917.45	±	19.80	^h^
A7	206.12	±	0.78	^m^	2430.00	±	30.45	^n^
A1f	485.27	±	2.52	^j^	4838.45	±	25.68	^i^
A2f	747.22	±	7.58	^f^	9495.92	±	56.89	^b^
A3f	980.48	±	3.16	^b^	8142.92	±	123.25	^c^
A4f	314.28	±	2.04	^k^	3820.01	±	19.47	^k^
A5f	823.94	±	5.46	^e^	6026.11	±	70.03	^f^
A6f	563.94	±	3.18	^i^	3961.89	±	18.42	^j^
A7f	626.58	±	5.23	^h^	5177.77	±	69.51	^g^
A8	185.73	±	1.48	^n^	1320.73	±	19.00	^r^
A9	186.83	±	1.99	^n^	1379.55	±	10.24	^r^
A10	863.30	±	1.34	^d^	6615.52	±	67.07	^e^
A11	885.88	±	8.07	^c^	7628.97	±	46.76	^d^
C1v	282.18	±	0.88	^b^	2778.16	±	54.31	^a^
C2v	158.59	±	1.55	^j^	1658.71	±	11.18	^j^
C3v	195.93	±	1.18	^h^	1929.25	±	21.32	^h^
C4v	56.81	±	0.08	^p^	516.63	±	1.56	^s^
C2	149.38	±	0.61	^k^	1590.39	±	14.45	^k^
C3	173.87	±	0.09	^i^	1901.67	±	17.26	^h^
C4	126.21	±	0.80	^n^	1154.44	±	12.28	^o^
C5	105.66	±	0.25	^o^	868.39	±	7.73	^r^
C6	227.21	±	2.56	^e^	2106.01	±	28.11	^e^
C7	212.22	±	2.84	^f^	2163.01	±	13.88	^d^
C8	136.18	±	0.06	^lm^	1123.39	±	9.53	^op^
C9	135.37	±	1.82	^m^	1228.92	±	19.82	^n^
C10	321.47	±	2.29	^a^	2715.83	±	13.77	^b^
C11	158.72	±	1.32	^j^	1445.35	±	16.22	^l^
C12	196.15	±	1.64	^h^	1854.47	±	11.75	^i^
C13	242.78	±	0.64	^d^	2028.74	±	32.77	^f^
C14	149.44	±	1.67	^k^	1446.56	±	24.67	^l^
C15	200.54	±	0.88	^g^	1997.30	±	39.10	^g^
C9v	107.29	±	0.62	^o^	955.25	±	4.85	^q^
C10N	273.47	±	2.26	^c^	2324.37	±	42.36	^c^
C16	172.24	±	0.25	^i^	1345.44	±	6.11	^m^
C17	138.27	±	0.23	^l^	1106.23	±	10.99	^p^

**Table 6:** The minimum inhibitory concentration (MIC) of the acacia and chestnut honey samples against pathogenic Gram positive and Gram-negative bacteria.

Sample/Pathogenic Bacteria	A8	A9	C1v	C2	C4v	C4	C8	C9v	C16	C17
	2015		2013				2014		2015	
*Listeria monocytogenes* ATCC 13932	50%	50%	0%	0%	0%	0%	0%	0%	12.5%	12.5%
*Listeria monocytogenes*—food isolate	50%	50%	0%	0%	0%	0%	0%	0%	50%	25%
*Salmonella* ser. Enteritidis ATCC 13076	50%	50%	0%	50%	0%	50%	25%	50%	50%	12.5%
*Salmonella* ser. Enteritidis—food isolate	50%	50%	0%	50%	0%	50%	50%	50%	50%	12.5%
*Yersinia enterocolitica* ATCC 23715	50%	25%	0%	0%	0%	0%	12.5%	12.5%	6.25%	6.25%
*Yersinia enterocolitica*—food isolate	50%	50%	0%	0%	0%	0%	6.25%	6.25%	6.25%	6.25%
*Bacillus cereus* ATCC 11778	6.25%	3.13%	25%	25%	6.25%	6.25%	6.25%	3.13%	3.13%	1.56%
*Bacillus cereus*—food isolate	50%	50%	50%	50%	50%	50%	25%	50%	50%	50%
*Pseudomonas aeruginosa* ATCC 27853	50%	50%	50%	50%	50%	50%	50%	50%	50%	25%
*Pseudomonas aeruginosa*—food isolate	50%	50%	50%	50%	50%	50%	50%	50%	50%	25%
*Escherichia coli* ATCC 25922	50%	50%	50%	50%	50%	50%	25%	50%	50%	12.5%
*Escherichia coli*—food isolate	50%	50%	50%	50%	50%	50%	25%	50%	50%	12.5%
*Staphylococcus aureus* ATCC 25923	50%	12.5%	50%	25%	12.5%	12.5%	6.25%	12.5%	6.25%	6.25%
*Staphylococcus aureus*—food isolate	25%	12.5%	50%	50%	12.5%	50%	6.25%	6.25%	6.25%	6.25%

## Data Availability

Data are available on request.

## Supplementary Materials

The following supporting information can be downloaded at https://www.mdpi.com/article/10.3390/molecules30040919/s1, Table S1. Element content of Croatian (a) acacia and (b) chestnut honey. Table S2. Pearson’s correlation coefficient between the polyphenolic content and the antioxidant activity (FRAP) of Croatian (a) acacia and (b) chestnut honey. Marked correlations are significant at *p* < 0.05. Api = apigenin, Benz = benzoic acid, Chl = chlorogenic acid, Chr = chrysin, Cin = cinnamic acid, Coum = p-coumaric acid, Dai = 3′,4′,7-trihydroxyisoflavone (3′-hydroxydaidzein), Fer = ferulic acid, FRAP = ferric-reducing/antioxidant power assay, Gal = gallic acid, Gin = galangin, Hes = hesperetin, K = kaempferol, Pbs = pinobanksin, Pcm = pinocembrin, Prc = protocatechuic acid, Q = quercetin, Syr = syringic acid, TiFs = total identified flavonoids, TiPs = total identified phenolic compounds, TiPAs = total identified phenolic acids and, TPs = total phenols, Van = vanillic acid. Table S3. Pearson’s correlation coefficient between the detected elements in Croatian (a) acacia and (b) chestnut honey. Marked correlations are significant at *p* < 0.05. Table S4. Characterization of Croatian (a) acacia honey collected in 2013 and 2015, and (b) chestnut honey samples collected in 2013, 2014 and 2015. HMF = hydroxymethylfurfural content, MPC = minimum permissible concentration, *DN = mL 1% starch solution per g of honey/h at 40 °C. Table S5. Names and descriptions of the honey samples. RT = room temperature, N = normal extension (part of the hive), s = small extension (part of the hive), * = repeated sample triplicate, CF = centrifuged, NCF = non-centrifuged. Table S6. Calibration curves and the R2 values of the phenolic acids and flavonoids for the HPLC analysis.

## Abbreviations

Api = apigenin, Benz = benzoic acid, CF = centrifuged, Chl = chlorogenic acid, Chr = chrysin, Cin = cinnamic acid, Coum = 4-coumaric acid, Dai = 3′,4′,7-trihydroxyisoflavone (3′-hydroxydaidzein), Fer = ferulic acid, FRAP = ferric-reducing/antioxidant power assay, Gal = gallic acid, Gin = galangin, Hes = hesperetin, HPLC = high-performance liquid chromatography, K = kaempferol, N = normal extension (part of the hive), NCF = non-centrifuged, Pbs = pinobanksin, Pcm = pinocembrin, Prc = protocatechuic acid, Q = quercetin, RT = room temperature, s = small extension (half-super; part of the hive), Syr = syringic acid, TiFs = total identified flavonoids, TiPs = total identified phenolic compounds, TiPAs = total identified phenolic acids, TPs = total phenols, Van = vanillic acid, TXRF = total reflection X-ray fluorescence
