# Supplementary material for: Influence of Season, Hive Position, Extraction Method and Storage Temperature on Polyphenols and Antioxidant Activity of Croatian Honey
Source: Molecules. 2025 Feb 17;30(4):919. doi: 10.3390/molecules30040919 (PMC11858134; doi:10.3390/molecules30040919)
Supplement: Supplementary file 1 [file molecules-30-00919-s001.zip › molecules-3424868-supplementary.pdf]

molecules

MDPI

Table S1. Element content of Croatian (**a**) acacia and (**b**) chestnut honey.

(**a**)	A	A1	A2	A3	A4s	A4s*	A5	A6	A7	A8	A9	A10	A11
Element	mg/kg	mg/kg	mg/kg	mg/kg	mg/kg	mg/kg	mg/kg	mg/kg	mg/kg	mg/kg	mg/kg	mg/kg	mg/kg
P	3.2	4.1	2.9	3.1	2.5	1.4	4.9	3.7	3.9	2.6	20.8	2.7	0.0
S	0.0	0.0	0.0	0.0	0.0	0.0	0.0	0.0	0.0	0.0	0.0	0.0	0.0
Cl	1.7	1.7	0.5	1.7	1.7	2.5	1.2	2.7	3.4	0.4	1.6	0.7	0.0
K	55.7	37.1	27.9	40.5	46.7	63.5	47.9	71.9	79.6	44.1	53.5	27.2	25.7
Ca	10.0	6.3	3.7	7.3	8.4	10.0	7.5	10.2	15.2	5.8	9.8	10.6	4.4
Cr	0.1	0.0	0.0	0.2	0.0	0.3	0.1	0.1	0.1	0.0	1.1	0.1	0.0
Mn	0.2	0.0	0.2	0.0	0.1	0.3	0.4	0.1	0.2	0.3	0.2	0.1	0.1
Fe	0.3	0.4	0.4	0.8	0.3	0.5	0.4	0.4	0.4	0.2	1.5	0.5	0.5
Ni	0.0	0.0	0.0	0.1	0.0	0.0	0.0	0.0	0.0	0.0	0.0	0.0	0.0
Cu	0.4	0.4	0.3	0.3	0.3	0.4	0.3	0.4	0.4	0.3	1.7	0.3	0.5
Zn	0.1	0.1	0.2	0.2	0.2	0.2	0.2	0.2	0.2	0.2	0.2	0.3	0.1
Br	0.5	0.0	0.0	0.6	0.0	0.0	3.2	0.1	0.1	0.2	0.1	0.2	0.1
Rb	0.1	0.0	0.0	0.0	0.1	0.2	0.0	0.1	0.2	0.1	0.1	0.0	0.0
Sr	0.0	0.0	0.0	0.0	0.0	0.0	0.0	0.0	0.0	0.0	0.0	0.0	0.0
Pb	0.1	0.0	0.0	0.0	0.1	0.0	0.0	0.0	0.1	0.1	0.0	0.0	0.0

molecules MDPI

(**b**) Element	C1v mg/kg	C2v mg/kg	C3v mg/kg	C4v mg/kg	C2 mg/kg	C3 mg/kg	C4 mg/kg	C5 mg/kg	C6 mg/kg	C7 mg/kg	C8 mg/kg	C9 mg/kg	C11 mg/kg	C12 mg/kg	C13 mg/kg	C14 mg/kg	C15 mg/kg	C10N mg/kg	C16 mg/kg	C17 mg/kg
P	1.9	4.0	6.6	3.8	5.4	3.3	2.0	2.8	1.4	3.3	4.0	1.4	4.0	1.6	3.2	4.3	3.1	3.1	3.3	2.9
S	0.0	0.0	0.0	0.0	0.0	0.0	0.0	0.0	0.0	0.0	0.0	0.0	0.6	0.0	0.0	0.0	1.0	0.0	0.0	1.9
Cl	3.6	3.6	4.6	3.8	5.6	3.4	3.7	2.8	1.5	3.4	5.3	4.6	3.6	6.0	6.4	5.0	5.0	8.7	3.3	7.1
K	521.1	616.8	525.0	496.8	846.8	583.5	538.4	623.2	283.3	658.2	520.0	590.6	376.3	513.8	630.7	699.7	429.0	774.7	324.6	403.8
Ca	49.5	57.4	53.2	46.2	73.4	58.3	58.2	62.0	32.1	54.5	50.2	48.4	50.0	58.4	55.8	64.1	43.4	76.0	31.7	88.7
Cr	0.1	0.0	0.0	0.0	0.0	0.1	0.0	0.0	0.0	0.0	0.0	0.0	0.0	0.0	0.0	0.0	0.0	0.0	0.0	2.1
Mn	7.4	8.9	7.9	5.9	10.4	8.5	8.6	8.8	4.0	7.9	5.8	6.8	4.9	5.5	5.6	7.2	4.5	6.7	4.7	4.5
Fe	0.5	0.4	0.5	0.7	0.4	0.6	0.4	0.7	0.4	0.4	0.7	0.3	0.4	0.5	0.3	0.5	0.4	0.7	1.1	2.4
Ni	0.0	0.0	0.1	0.0	0.0	0.0	0.0	0.0	0.0	0.0	0.2	0.0	0.0	0.0	0.1	0.0	0.1	0.0	0.1	0.0
Cu	0.4	0.5	0.5	0.5	0.4	0.4	0.4	0.5	0.4	0.5	0.4	0.4	0.4	0.4	0.4	0.9	0.4	0.4	0.5	0.6
Zn	0.2	0.3	0.4	0.3	0.4	0.3	0.2	0.4	0.2	0.3	0.5	0.3	0.7	0.3	0.3	0.4	0.3	0.6	0.6	0.8
Br	0.2	0.2	0.2	0.2	0.4	0.4	0.6	0.2	23.4	0.5	0.3	0.2	0.2	0.1	0.2	0.1	0.2	0.2	0.4	0.8
Rb	5.6	6.6	6.4	3.9	7.0	6.5	6.7	6.8	2.8	6.0	4.4	4.9	2.9	3.6	3.7	4.8	3.5	4.0	2.8	2.9
Sr	0.0	0.1	0.2	0.0	0.2	0.1	0.2	0.2	0.0	0.2	0.0	0.0	0.0	0.1	0.0	0.0	0.0	0.1	0.0	0.0
Pb	0.1	0.1	0.2	0.1	0.1	0.2	0.1	0.2	0.1	0.1	0.1	0.2	0.1	0.1	0.1	0.1	0.1	0.1	0.1	0.1

molecules

MDPI

Table S2. Pearson's correlation coefficient between the polyphenolic content and the antioxidant activity (FRAP) of Croatian (**a**) acacia and (**b**) chestnut honey. Marked correlations are significant at $p < 0.05$. Api = apigenin, Benz = benzoic acid, Chl= chlorogenic acid, Chr = chrysin, Cin = cinnamic acid, Coum = 4-coumaric acid, Dai = 3,4,7-trihydroxyizoflavon (3'-hydroxydaidzein), Fer = ferulic acid, FRAP = ferric-reducing/antioxidant power assay, Gal = gallic acid, Gin = galangin, Hes = hesperetin, K = kaempferol, Pbs = pinobanksin, Pcm = pinocembrin, Prc = protocatechuic acid, Q = quercetin, Syr = syringic acid, TiFs = total identified flavonoids, TiPs = total identified phenolic compounds, TiPAs = total identified phenolic acids and, TPs = total phenols, Van = vanillic acid.

(a)	TiP	TiPA	TiF	TP	FRAP	Gal	Benz	Van	Chl	Syr	Coum	Fer	Cin	Pbs	Q	K	Api	Pcm	Chr	Gin
TiPs	1.00																			
TiPAs	**0.99**	1.00																		
TiFs	**0.72**	**0.59**	1.00																	
TPs	0.18	0.21	0.01	1.00																
FRAP	0.21	0.24	0.01	**0.99**	1.00															
Gal	**0.71**	**0.77**	0.20	0.22	0.21	1.00														
Benz	0.17	0.17	0.09	-0.07	-0.04	-0.11	1.00													
Van	0.39	0.47	-0.10	0.22	0.26	**0.50**	-0.04	1.00												
Chl	**0.87**	**0.80**	**0.87**	0.09	0.12	0.29	0.26	0.08	1.00											
Syr	**0.64**	**0.60**	**0.60**	0.00	-0.02	0.44	0.27	-0.05	**0.61**	1.00										
Coum	**0.77**	**0.77**	**0.51**	0.25	0.32	0.29	0.16	0.45	**0.80**	0.18	1.00									
Fer	**0.74**	**0.73**	**0.50**	0.10	0.18	0.24	0.15	0.45	**0.78**	0.16	**0.98**	1.00								
Cin	0.29	0.21	**0.53**	-0.15	-0.15	0.02	0.02	-0.02	0.41	0.13	0.22	0.21	1.00							
Pbs	**0.65**	**0.52**	**0.97**	0.01	0.00	0.13	0.12	-0.21	**0.85**	**0.69**	0.42	0.40	**0.50**	1.00						
Q	-0.11	-0.03	-0.41	-0.17	-0.14	0.20	-0.43	**0.54**	-0.37	-0.19	-0.06	0.02	-0.40	-0.48	1.00					
K	**0.65**	**0.57**	**0.80**	-0.11	-0.06	0.04	0.28	-0.07	**0.88**	0.28	**0.73**	**0.76**	**0.54**	**0.74**	-0.41	1.00				
Api	**0.65**	**0.52**	**0.96**	-0.04	-0.03	0.06	0.15	-0.18	**0.88**	**0.53**	**0.53**	**0.53**	**0.54**	**0.96**	-0.46	**0.86**	1.00			
Pcm	**0.58**	0.46	**0.91**	-0.05	-0.03	0.10	0.10	-0.06	**0.72**	0.33	0.48	**0.50**	**0.54**	**0.81**	-0.36	**0.81**	**0.86**	1.00		
Chr	**0.80**	**0.74**	**0.74**	0.19	0.17	**0.61**	-0.05	0.32	**0.65**	**0.50**	**0.52**	**0.49**	0.29	**0.61**	-0.01	0.46	**0.57**	**0.69**	1.00	
Gin	**0.66**	**0.53**	**0.98**	0.00	0.00	0.13	0.03	-0.12	**0.83**	0.46	**0.53**	**0.52**	**0.57**	**0.93**	-0.45	**0.83**	**0.95**	**0.95**	**0.70**	1.00

Bold marked correlations are significant at $p < 0.05$

molecules

MDPI

**(b)**	TiP	TiPA	TiF	TP	FRAP	Prc	Benz	Van	Syr	Fer	Dai	Cin	Pbs	Q	Hes	K	Api	Pcm	Chr	Gin
TiPs	1.00																			
TiPAs	**0.99**	1.00																		
TiFs	**0.60**	**0.48**	1.00																	
TPs	0.02	0.01	0.08	1.00																
FRAP	0.01	0.00	0.03	**0.96**	1.00															
Prc	**0.71**	**0.76**	0.10	-0.07	-0.06	1.00														
Benz	**0.73**	**0.70**	**0.57**	-0.27	-0.27	**0.45**	1.00													
Van	**0.62**	**0.58**	**0.60**	-0.02	-0.06	0.02	**0.71**	1.00												
Syr	**0.77**	**0.73**	**0.63**	0.06	0.01	0.15	**0.66**	**0.94**	1.00											
Fer	**0.61**	**0.59**	0.39	0.24	0.24	0.13	0.15	0.35	**0.58**	1.00										
Dai	0.34	0.31	0.33	-0.24	-0.10	-0.03	0.42	**0.44**	0.42	0.37	1.00									
Cin	-0.01	0.01	-0.12	-0.20	-0.02	-0.15	-0.02	-0.04	-0.02	0.37	**0.62**	1.00								
Pbs	0.35	0.29	**0.55**	0.41	0.21	0.06	0.11	0.33	0.40	0.29	-0.30	**-0.44**	1.00							
Q	-0.17	-0.19	-0.01	0.15	0.12	-0.05	-0.20	-0.26	-0.24	-0.10	**-0.43**	-0.32	0.11	1.00						
Hes	0.30	0.24	**0.52**	-0.02	0.10	0.21	**0.48**	0.17	0.10	0.02	0.29	0.07	-0.16	-0.09	1.00					
K	-0.08	-0.04	-0.28	0.12	0.29	0.20	-0.09	-0.42	-0.37	0.01	0.13	**0.50**	-0.41	-0.33	0.40	1.00				
Api	0.40	0.30	**0.75**	-0.15	-0.20	-0.13	0.39	**0.62**	**0.63**	0.30	**0.45**	0.03	0.37	-0.10	0.06	**-0.54**	1.00			
Pcm	**0.50**	0.40	**0.80**	-0.10	-0.21	-0.06	**0.43**	**0.66**	**0.71**	0.40	0.24	-0.12	**0.53**	-0.01	0.03	**-0.64**	**0.91**	1.00		
Chr	0.39	0.29	**0.76**	0.12	-0.02	0.08	0.30	0.32	0.40	0.23	-0.14	**-0.48**	**0.63**	0.21	0.17	**-0.47**	**0.52**	**0.72**	1.00	
Gin	0.35	0.25	**0.76**	0.19	0.01	0.00	0.18	0.32	0.41	0.28	-0.14	-0.40	**0.85**	0.19	-0.03	**-0.52**	**0.66**	**0.79**	**0.87**	1.00

Bold marked correlations are significant at $p < 0.05$

molecules

MDPI

Table S3. Pearson's correlation coefficient between the detected elements in Croatian (**a**) acacia and (**b**) chestnut honey. Marked correlations are significant at $p < 0.05$.

(**a**)	P	Cl	K	Ca	Cr	Mn	Fe	Ni	Cu	Zn	Br	Rb	Pb
P	1.00												
Cl	0.12	1.00											
K	0.18	**0.90**	1.00										
Ca	0.21	**0.80**	**0.78**	1.00									
Cr	**0.92**	0.18	0.23	0.27	1.00								
Mn	0.11	-0.04	0.28	0.08	0.13	1.00							
Fe	**0.86**	0.05	0.01	0.12	**0.93**	-0.12	1.00						
Ni	-0.07	0.05	-0.13	-0.11	0.04	-0.43	0.27	1.00					
Cu	**0.94**	0.05	0.14	0.15	**0.94**	0.05	**0.89**	-0.13	1.00				
Zn	0.12	0.05	0.04	0.33	0.16	0.18	0.14	0.08	-0.03	1.00			
Br	0.02	-0.11	-0.01	-0.07	-0.06	0.54	-0.08	0.07	-0.15	0.07	1.00		
Rb	0.10	**0.70**	**0.84**	**0.69**	0.24	0.35	-0.02	-0.28	0.16	0.08	-0.31	1.00	
Pb	-0.17	0.20	0.36	0.33	-0.26	0.18	-0.44	-0.19	-0.20	-0.12	-0.15	0.52	1.00

Bold marked correlations are significant at $p < 0.05$

(b)	P	S	Cl	K	Ca	Cr	Mn	Fe	Ni	Cu	Zn	Br	Rb	Sr	Pb
P	1.00														
S	-0.04	1.00													
Cl	0.15	0.31	1.00												
K	0.31	-0.38	0.43	1.00											
Ca	0.20	0.38	**0.68**	**0.58**	1.00										
Cr	-0.08	**0.84**	0.35	-0.24	**0.57**	1.00									
Mn	0.35	**-0.46**	-0.09	**0.76**	0.37	-0.28	1.00								
Fe	-0.02	**0.73**	0.32	-0.30	**0.46**	**0.91**	-0.33	1.00							
Ni	0.32	-0.01	0.14	-0.22	-0.33	-0.14	-0.31	0.00	1.00						
Cu	0.29	0.14	0.04	0.13	0.26	0.26	0.04	0.30	-0.15	1.00					
Zn	0.30	**0.57**	**0.45**	-0.14	0.41	**0.56**	-0.33	**0.69**	0.14	0.22	1.00				
Br	-0.34	-0.07	-0.43	-0.44	-0.40	-0.03	-0.35	-0.09	-0.13	-0.13	-0.26	1.00			
Rb	0.30	-0.43	-0.21	**0.63**	0.26	-0.27	**0.96**	-0.35	-0.21	0.00	-0.43	-0.30	1.00		
Sr	0.30	-0.31	-0.07	**0.51**	0.34	-0.20	**0.77**	-0.21	-0.23	-0.11	-0.20	-0.18	**0.77**	1.00	
Pb	0.10	-0.19	-0.22	0.12	0.00	-0.10	0.36	-0.10	-0.04	-0.07	-0.12	-0.12	**0.46**	0.33	1.00

Bold marked correlations are significant at $p < 0.05$

molecules

MDPI

Table S4. Characterization of Croatian (**a**) acacia honey collected in 2013 and 2015 and (**b**) chestnut honey samples collected in 2013, 2014 and 2015. HMF = hydroxymethylfurfural content, MPC = minimum permissible concentration, *DN = mL 1% starch solution per g of honey/h at 40°C.

(**a**)	Units	MPC	Year	
			2013	2015
Pelud *Robinia pseudoacacia*	%	>20%	21.0	37.0
Reduced sugars	g/100 g	>60	64.7	
Sucrose	g/100 g	<5	1.4	
Electrical conductivity	mS/cm	>0.8	0.2	0.1
Free acids	mEq/100 g	<50	12.4	
Diastasis activity	DN*	>8	18.0	12.8
HMF	mg/kg	<40	1.4	0.2
Water	%	<20	18.0	16.9
Activity concentration ^{137}Cs	Bq/kg	>1000	0.6	0.1
Activity concentration ^{40}K	Bq/kg		54.0	11.9
Aerobic mesophilic bacteria/g - incubation at 30°C	cfu/g	1000	<10	
Enterobacteriaceae/g - incubation at 37°C	cfu/g	10	<10	
Sulfite-reducing clostridium/g	cfu/g	10	<10	
Yeasts and mold/g	cfu/g	10	<10	
Antibiotics		poz / neg	neg	

(**b**)	Units	MPC	Year		
			2013	2014	2015
Castanea sativa pollen	%	>85%	87.0	92.0	89.1
Reduced sugars	g/100 g	>60	62.8	65.2	
Sucrose	g/100 g	<5	0.8	3.5	
Electrical conductivity	mS/cm	>0.8	1.8	1.3	1.1
Free acids	mEq/100 g	<50	13.8	13.5	
Diastasis activity	DN*	>8	29.0	25.0	28.1
HMF	mg/kg	<40	2.6	0.1	1.3
Water	%	<20	19.1	18.5	19.2
Activity concentration ^{137}Cs	Bq/kg	>1000		3.1	0.8
Activity concentration ^{40}K	Bq/kg			266.9	56.9
Aerobic mesophilic bacteria/g - incubation at 30°C	cfu/g	1000	<10	<10	
Enterobacteriaceae/g - incubation at 37°C	cfu/g	10	<10	<10	
Sulfite-reducing clostridium/g	cfu/g	10	<10	<10	
Yeasts and mold/g	cfu/g	10	<10	<10	
Antibiotics		poz / neg	neg	neg	

Table S5. Names and descriptions of the honey samples. Hive numbers 1–5 and 40–44 denote edge hives, while numbers 6–39 denote hives in the middle. RT = room temperature, N = normal extension (part of the hive), s = small extension = half-super (part of the hive), * = repeated sample triplicate, CF = centrifuged, NCF = non-centrifuged.

Sample name	Description		Year
A	Acacia	Aggregation sample from 2013	2013
A1	Acacia	Aggregation sample from 2013, RT	
A2	Acacia	Hive No. 14, N, NCF	
A3	Acacia	Hive No. 44, s, NCF	
A4	Acacia	Hive No. 32, N, NCF	
A4s	Acacia	Hive No. 32, s, NCF	
A4s*	Acacia	Hive No. 32, s, NCF	
A5	Acacia	Hive No. 22, N, NCF	
A6	Acacia	Hive No. 42, NCF	
A7	Acacia	Hive No. 32, N, CF	
A1f	Acacia	Aggregation sample from 2013, 4 °C	
A1f*	Acacia	Aggregation sample from 2013, 4 °C	
A2f	Acacia	Hive No. 14, N, NCF	
A3f	Acacia	Hive No. 44, PN, NCF	
A4f	Acacia	Hive No. 2, N, NCF	
A5f	Acacia	Hive No. 22, N, NCF	
A6f	Acacia	Hive No. 42, NCF	
A7f	Acacia	Hive No. 32, N, CF	
A7f*	Acacia	Hive No. 32, N, CF	
A8	Acacia	Aggregation sample from 2015 RT	2015
A9	Acacia	Hive No. 3, NCF	
A10	Acacia	Hive No. 2, NCF	
A11	Acacia	Hive No. 5, NCF	
C1v	Chestnut	Aggregation sample from 2013 RT	2013
C2v	Chestnut	Hive No. 44, CF	
C3v	Chestnut	Hive No. 22, CF	

C4v	Chestnut	Hive No. 23, CF (Nylon)	
C2	Chestnut	Hive No. 44, NCF	
C3	Chestnut	Hive No. 22, NCF	
C4	Chestnut	Hive No. 23, NCF	
C5	Chestnut	Hive No. 14, NCF	
C6	Chestnut	Hive No. 36, NCF	
C7	Chestnut	Hive No. 2, NCF	
C8	Chestnut	Aggregation sample from 2014 RT	2014
C9	Chestnut	Hive No. 5, NCF	
C10	Chestnut	Hive No. 4, s, NCF	
C11	Chestnut	Hive No. 5, s, NCF	
C12	Chestnut	Hive No. 6, s, NCF	
C13	Chestnut	Hive No. 2, s, NCF	
C14	Chestnut	Hive No. 1, s, NCF	
C15	Chestnut	Hive No. 5, s, NCF	
C9v	Chestnut	Hive No. 5, CF	
C10N	Chestnut	Hive No. 4, N, NCF	
C16	Chestnut	Aggregation sample from 2015 RT	2015
C17	Chestnut	Hive No. 5, CF	

Table S6. Calibration curves and the R^2 values of the phenolic acids and flavonoids for the HPLC analysis.

No.	Standard compound	Equation	R^2
1	Gallic acid	y = 2131.2x - 28.918	0.9994
2	Protocatechuic acid	y = 890.02x - 1.0559	0.9990
3	Benzoic acid	y = 948.65x + 2.5952	0.9988
4	Vanillic acid	y = 1790.2x + 0.3719	1.0000
5	Chlorogenic acid	y = 711.68x - 36.438	0.9950
6	Caffeic acid	y = 1924.9x - 12.865	0.9994
7	Syringic acid	y = 1002.8x + 0.003	0.9989
	caffein	y = 1510.9x - 1.4982	0.9997
8	4-coumaric acid	y = 2933.2x + 6.8614	0.9988
9	Ferulic acid	y = 2056.5x + 1.4603	0.9991
10	Salicylic acid		
11	3,4,7-trihidroxyisoflavon (3'-hydroxydaidzein)	y = 1074.5x - 4.5826	0.9992
12	Cinnamic acid	y = 6397.7x + 11.52	0.9990
13	Pinobanksin	y = 1670.9x + 1.6348	0.9986
14	Quercetin	y = 433.95x - 8.2383	0.9978
	genistein	y = 1627.5x - 0.3088	0.9994
15	Hesperetin	y = 501.53x + 0.5735	0.9994
16	Kaempferol	y = 845.33x - 10.434	0.9984
17	Apigenin	y = 841.56x - 3.1395	0.9994
18	Pinocembrin	y = 1534.8x + 2.5901	0.9988
19	Chrysin	y = 2941.4x - 2.0298	0.9993
20	Galangin	y = 818.2x + 3.6117	0.9993
